# The effect of relative pitch size on physiological, physical, technical and tactical variables in small-sided games: a literature review and practical guide

**DOI:** 10.3389/fspor.2025.1592536

**Published:** 2025-05-06

**Authors:** Michael C. Rumpf, Johannes Jäger, Filipe M. Clemente, Stefan Altmann, Matthias Lochmann

**Affiliations:** ^1^ Department of Sport Science and Sport, Friedrich-Alexander University Erlangen-Nürnberg, Erlangen, Germany; ^2^ Football Performance Institute, footballscience.net, Dreieich, Germany; ^3^ Sports Performance Research Institute New Zealand, Auckland University of Technology, Auckland, New Zealand; ^4^ Escola Superior Desporto e Lazer, Instituto Politécnico de Viana do Castelo, Viana do Castelo, Portugal; ^5^ Gdansk University of Physical Education and Sport, Gdańsk, Poland; ^6^ Sport Physical Activity and Health Research & Innovation Center, Viana do Castelo, Portugal; ^7^ Institute of Sports and Sports Science, Karlsruhe Institute of Technology, Karlsruhe, Germany; ^8^ TSG ResearchLab GmbH, Zuzenhausen, Germany

**Keywords:** soccer, training, performance parameters, game formats, football

## Abstract

**Introduction:**

Manipulating relative pitch size is a common practice in soccer training; however, published evidence on its true effects for practical coaching purposes has been lacking in the scientific literature. This review aimed to identify and quantify changes in physiological, physical, technical, and tactical variables resulting from increases in relative pitch size during small-sided games (SSGs) in soccer.

**Methods:**

A literature search was conducted, resulting in the inclusion of 56 articles in this review. Linear regression analysis was performed to examine the total and percentage changes in relative pitch size and its influence on physiological, physical, technical, and tactical variables collected during SSGs.

**Results:**

Linear regression revealed that an increase in relative pitch size was significantly positively associated with higher values in lactate, RPE, and Edwards’ TRIMP (*p* < 0.05). No significant associations were found for heart rate metrics or player loads. Several physical variables showed significant positive associations (total distance, relative total distance, maximum speed, number of sprints, sprinting, high-speed running, jogging, acceleration and deceleration, high-metabolic load distance, work-to-rest ratio) and negative associations (walking distance) with increases in relative pitch size (*p* < 0.05). Larger relative pitch sizes were associated with a lower frequency of most technical actions (ball touches, dribbles, turnovers), while tactical variables showed significant positive associations (team width, surface area, stretch index, inter-team distance) and a negative association (spatial exploration distance) with changes in pitch size.

**Conclusion:**

Larger relative pitch sizes can be implemented as a task constraint in SSGs to enhance physiological (e.g., lactate, RPE), physical (e.g., total distance, number of sprints), and tactical variables (e.g., surface area). Caution should be exercised regarding the potential negative impact on technical outcomes when increasing relative pitch size. Practitioners in soccer can utilize the findings of this review to achieve desired effects by adjusting relative pitch size to target specific training outcomes.

## 1 Introduction

Small-sided games (SSGs) are a popular training tool widely used across all ages and skill levels for different purposes (1). Various reviews in the scientific literature have investigated different regulations and rules—so-called task constraints—during SSGs that influence physiological, physical, technical, and tactical parameters (2). For example, limited physiological parameters were reported in 2011, indicating that an increase in relative pitch size led to an increase in heart rate (HR), ratings of perceived exertion (RPE), and blood lactate concentration (1). However, also the concurrent reduction in player number and increase in relative pitch size elicit higher exercise intensity (1). An additional brief review (3) and a methodological proposal (4) in 2014 indicated somehow conflicting results. That is, smaller SSGs, i.e., 3 vs. 3, compared to 5 vs. 5, resulted in higher HR (3). Nevertheless, when player number were kept constant, a larger playing area increased the intensity of the SSG with a smaller playing area having the opposite effect (3). Maximum heart rate (HRmax), as well as lactate and RPE increased with fields of larger dimensions (4). Consequently, the increase in dependent variables might be a result of the combination of number of players in relation to pitch size. Indeed, two additional reviews in 2022 (5, 6) confirmed the effect of relative pitch size on physiological parameters concluding that larger pitch size (6) as well as relative areas per player increase mean heart rate (HRmean) (5) and RPE (6). Similarly, physical parameters were reported to increase with an increase in relative pitch size. More precisely, total distance covered (5, 6) as well as high-speed running (5, 6) increased with larger relative pitch size, whilst no differences were found with regards to acceleration and decelerations (6). However, it was also reported that smaller games showed greater overall distance, but also less jogging and walking distance (3) compared to larger games with more players. Consequently, the conflicting results might, yet again, derive from a change in relative pitch size vs. a change in pitch size.

Technical parameters were also investigated throughout the scientific literature and displayed seemingly more heterogenous results with amendments in pitch sizes. More tackling, dribbling, goal attempts and passes were reported in smaller games compared to larger games (3). However, also no differences were observed in passes and dribbles (6). Further research concluded that a reduction in the number of players and in the size of the pitch area increased the total ball contact per player and, therefore, the number of technical actions (7). Tactical observations were relatively limited in the scientific literature, yet they were also investigated in relation to changes in pitch size. A change in field length contributed to an increase in the team's length and the distance between teams (8). Furthermore, greater values in stretch index (i.e., dispersion of players in relation to the team's centroid) and surface area (i.e., covered space by the players of a team) have been observed (6), suggesting that players utilize more space as pitch size increases. However, no differences were found in positional centroid, regardless of format and age group (6).

The scientific problem in many of the aforementioned studies is that they simultaneously alter both pitch size and the number of players, leading to confounding effects in the results. This makes it difficult to determine whether observed changes in physiological, physical, technical, and tactical variables are solely due to pitch size or the interaction between pitch size and player numbers. That is, changing both player numbers and pitch size simultaneously leads to results that cannot be solely attributed to a change in one variable or the other. Consequently, it has been suggested that future studies should consider relative pitch size when comparing data across different small-sided game configurations to avoid confounding variables (5). Furthermore, coaches can use relative pitch size calculations to predict increases and decreases in expected training loads for different versions of SSGs (5). While studies have quantified changes in external load metrics (e.g., total distance, high-speed running) in relation to increases in relative pitch size (9, 10), they have not provided an in-depth analysis of additional variables across different performance dimensions, such as physiological, technical, and tactical aspects. As a result, there is a scarcity on scientific information as well as practical advice to coaches and practitioners with regards to the manipulation of the relative pitch size and its influence on physiological, physical, technical and tactical outcome variables. Such information could provide valuable guidance for planning SSGs in daily training, ensuring sustainable player development.

Therefore, the purpose of this review was to update and summarize the existing literature on SSGs in soccer, specifically regarding changes in relative pitch size and their effects on physiological, physical, technical, and tactical outcome variables. Additionally, this review aims to provide practical guidelines for each of these categories.

## 2 Methods

To gain an overview of the existing scientific literature on this research topic, a systematic literature search was conducted following the guidelines of the Preferred Reporting Items for Systematic reviews and Meta-Analyses (PRISMA) (11).

### 2.1 Eligibility criteria

This review followed the PICOS (Population, Intervention, Comparator, Outcomes, Study Design) approach: (P) soccer players of any age group, gender, or skill level, without injury, illness, or other clinical conditions; (I) smaller pitch sizes using any format of play (number of players involved) or other task conditions; (C) larger pitch sizes using any format of play (number of players involved) or other task conditions (keeping the same experimental conditions as in the smaller formats); (O) mean and standard deviation (SD) values for at least one of the following main outcomes: physiological responses, physical responses, technical actions, and tactical behaviors; and (S) observational longitudinal, cross-sectional, pilot, case studies, or experimental (non-) randomized controlled studies. For a detailed overview of the inclusion and exclusion criteria, please refer to Table 1.

**Table 1:** Inclusion and exclusion criteria (6).

Item by PICOS	Inclusion criteria	Exclusion criteria
Population	Soccer players from any age-group, gender, sex or skill, without injury, illness or other clinical condition.	Other sports than soccer
Intervention	• Each pitch size was tested at least twice with the same players (minimum of two repetitions). • The same absolute pitch dimensions were used while varying the number of players, leading to changes in relative pitch size. • When studies compared three or more pitch sizes under the same format or condition, the smallest pitch size was identified based on the lowest relative pitch size. • Experimental conditions remained identical across smaller and larger pitch sizes, ensuring consistency in team composition, playing regimen, and task constraints.	• The same pitch size was tested in only one repetition. • Smaller and larger pitch size conditions were not applied under the same contextual and experimental conditions (e.g., differences in team composition, playing regimen, or task constraints).
Outcome	At least one of the following category and outcome variable: • Physiological responses (e.g., heart rate) • Physical demands (e.g., total distance covered) • Technical execution (e.g., passes); • Tactical behavior (e.g., collective organization measures)	Other outcome measures than those related to immediate physiological, physical, technical and tactical responses (e.g., cognitive aspects during SSGs)
Study design	Observational longitudinal, cross-sectional, pilot, case studies, or experimental (non-) randomized controlled studies.	No observational longitudinal, cross-sectional, pilot, case studies, or experimental (non-) randomized controlled studies.
Additional criteria	Included studies were peer-reviewed, original, full-text articles or conference abstracts (provided they contained the necessary methodological details). Studies had to be written in English, Portuguese, or Spanish.	Written in a language other than the selected ones (English, Portuguese, and/or Spanish). Reviews, letters to the editor, trial registrations, protocol proposals, editorials, book chapters.

### 2.2 Information sources

Electronic databases, including PubMed, PsycINFO, Scielo, Scopus, SPORTDiscus, and Web of Science, were searched for relevant publications up to February 25, 2025. Additionally, the reference lists of the included studies retrieved were manually searched to identify potentially eligible studies not captured by the electronic searches. Duplicates were identified using a reference manager software (EndNoteTM X9, Clarivate Analytics, Philadelphia, PA, USA).

### 2.3 Search strategy

Keywords and synonyms were entered in various combinations in all fields using Boolean operators: “soccer”, “football”, “small-sided games”, “small-sided game”, “conditioned games”, “SSG”, “small-sided conditioned games”, “pitch” OR “field”. An overview of the search terms and applied filters for each database is provided in the supplemental material (Supplementary Table S1).

### 2.4 Selection process

The selection of relevant articles followed a two-step process. After removing duplicates, the first stage involved screening titles and abstracts by two reviewers (MR and JJ). In the second phase, full-texts were assessed based on predefined criteria. In cases of disagreement between the two reviewers, a third reviewer (ML) was included to resolve conflicts through discussion.

### 2.5 Data collection process

Data extraction was conducted by two independent reviewers (i.e., MR and JJ) using a standardized data extraction form using Microsoft Excel (Version 2108, Microsoft, Redmond, WA, USA). Both reviewers worked independently to minimize bias, and any discrepancies were resolved through discussion. To ensure accuracy and completeness, study authors were contacted when clarification or additional data were needed. For articles published in languages other than English, professional translation services and native-speaking collaborators were consulted to ensure accurate data extraction. Any uncertainties arising from translations were resolved through discussion among the review team.

### 2.6 Extraction and categorization of data

The primary outcomes of interest in this review were performance-related parameters during SSGs. Specifically, data were sought on physiological, physical, technical, and tactical parameters. In order to establish consistency in data analysis and reporting, only measures that were analyzed in two or more scenarios were included. For physiological responses, the following measures were extracted: maximal and average HR responses, as well as HRmax across different zones, were analyzed. Due to data heterogeneity, variables were categorized according to the following standardized thresholds: <75%, 75%–85%, and >85% of HRmax. Furthermore, lactate concentrations, RPE, Player Load, and Edwards' Training Impulse (TRIMP) were extracted.

For physical responses, the following measures were extracted: Total and relative distance covered, the number and distance of accelerations and decelerations, the number of sprints, distance at individual speed, maximal speed, and mechanical workload metrics—including metabolic load distance and metabolic load time (derived from inertial measurement units)—were examined. Additionally, change of direction frequency, work-to-rest ratio, and distance covered at various speed thresholds were analyzed. To account for the heterogeneity in speed zone classifications across studies, speed thresholds were standardized as followed: walking (0–9 km/h), jogging (7–14 km/h), running (14–20 km/h), high-speed running (18–21 km/h), and sprinting (>20 km/h).

For technical variables the following list of measures were extracted: passes (total and relative number), ball possessions, shots (total and relative number), turnovers, dribbles (total and relative number), and ball touches. For tactical outcome measures the following list of variables were extracted: team width/length, surface area, stretch index, inter-team distance, length per width ratio, and spatial exploration index. Due to the heterogeneity of data and variables reported in the individual publications, the variables were categorized into the aforementioned groups while maintaining a logical assignment. The categorization was conducted independently by two authors (i.e., MR and JJ). In cases of disagreement, a third author (i.e., ML) was consulted until a consensus was reached. A detailed overview of the variable types assigned to each category is provided in the Supplementary Material (Supplementary Table S2).

Mean and standard deviation (SD) for each outcome of the smaller and larger pitch sizes were collected. When raw data were unavailable in the included studies, an online tool (WebPlotDigitizer) was used to extract numerical values from published graphs. The data extraction process was conducted by the first author (MR) to ensure consistency and accuracy. The intra-rater reliability to extract data was 0.09%. Additionally, the following information was extracted from the included studies: sample characteristics (e.g., number of participants, competitive level, age); SSG format (e.g., 5 vs. 5, 6 vs. 6); pitch dimensions and relative pitch size; and intervention regimen (e.g., number and duration of bouts and/or games, recovery duration and modality) (Supplementary Table S3). The relative pitch size was calculated by dividing the total pitch size (i.e., length × width) by the number of players (excluding goalkeepers). The total and relative change in relative pitch size was determined by dividing the larger relative pitch size by 1% of the smaller relative pitch size and subtracting 100. Similarly, the total and percentage change in the outcome variables were calculated by dividing the mean value while playing on the larger relative pitch size by 1% of the mean value while playing on the smaller relative pitch size and subtracting 100. This resulted in a positive value when the outcome increased with increasing pitch size. Both the total and percentage change in the mean were then used in the statistical analysis.

### 2.7 Study risk of bias assessment

The risk of bias assessment was carried out using the Risk of Bias in Non-Randomized Studies of Interventions (ROBINS-I) tool (68), which was deemed suitable for the non-randomized observational studies in this review. The tool assesses the risk of bias across seven domains: (1) bias due to confounding, (2) bis in selection of participants into the study, (3) bias in classification of interventions, (4) bias due to deviations from intended interventions, (5) bias due to missing data, (6) bias in measurement of outcomes, (7) bias in selection of the reported results. For each study, an overall risk of bias judgment was made by summarizing the results across all domains. The judgment categories used were: *Low Risk*, *Moderate Risk*, *Serious Risk*, and *Critical Risk*. An overall judgment was assigned based on the pattern of risk across the domains. For example, if a study was assessed as having a high risk of bias in one or more domains (e.g., bias due to confounding or missing data), the overall judgment was classified as *High Risk* (68). Two independent reviewers (i.e., MR and JJ) conducted the risk of bias assessments. In cases where there were discrepancies between the reviewers' assessments, a third reviewer (i.e., ML) was involved to reach a consensus. This process was discussed until a final agreement was made, ensuring that the assessment was as accurate as possible.

### 2.8 Statistical analysis

Data were processed utilizing Microsoft Excel (version 16.78.3; Microsoft; Redmond, Washington, USA) and further analyzed with the IBM SPSS statistical software package (version 25.0; IBM Corporation, New York, USA). Descriptive variables were quantified, and data were reported using 95% confidence limits (CL) and means. Before conducting statistical analyses, assumptions for normality (Shapiro–Wilk test), homogeneity of variances (Levene's test), and linearity were assessed. A linear regression model and a one-way analysis of variance (ANOVA) were then utilized to establish significant changes in the dependent variable (i.e., physiological, physical, technical and tactical parameters) in relation to the independent variable (i.e., relative pitch size) (10). Finally, the regression equations of significant models were used to examine the changes in the outcome variable by varying the predictor values (x). The significance level (α) was set at 0.05.

## 3 Results

### 3.1 Study identification

**Figure 1:**
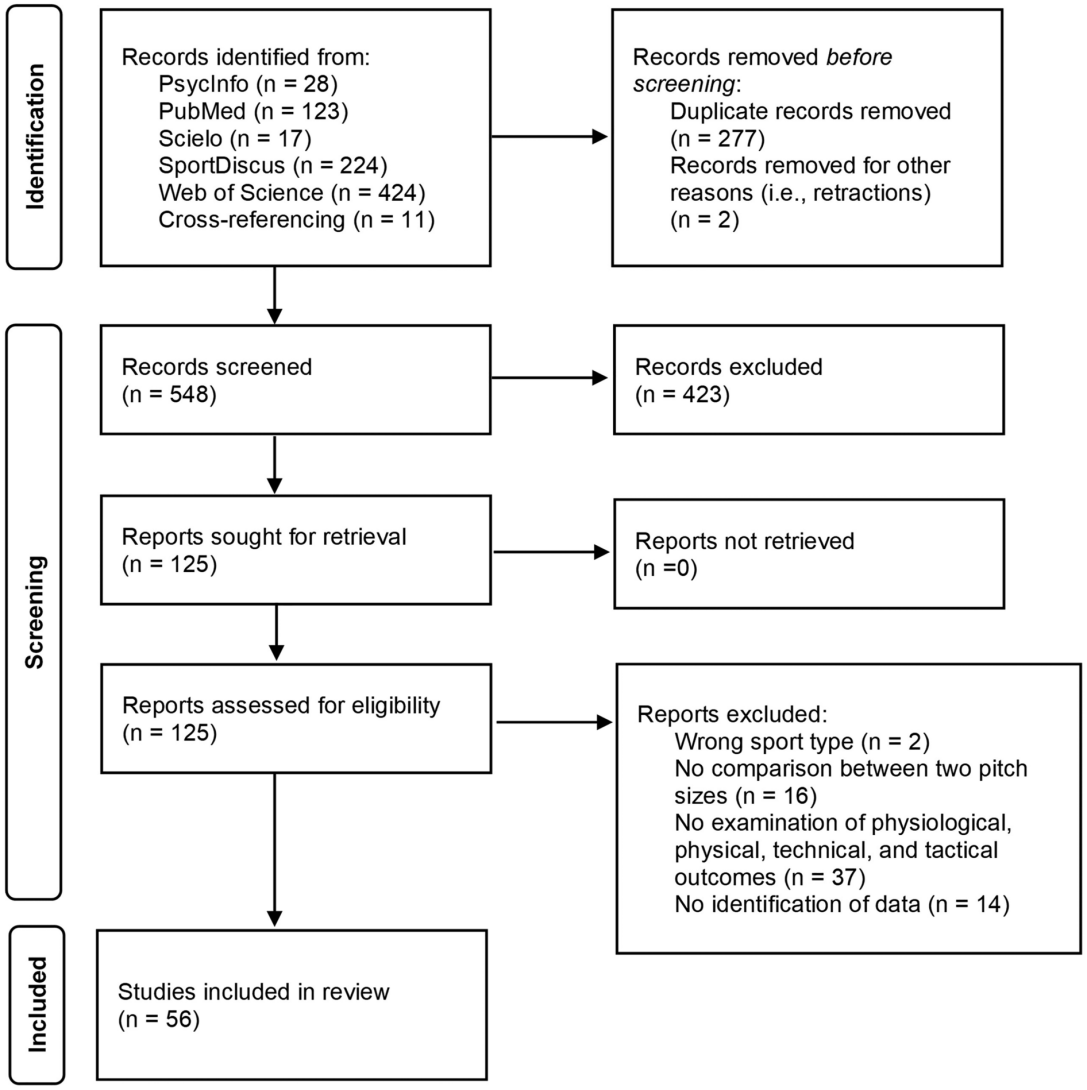
Flowchart of study selection.

The databases search identified an initial 828 titles. Duplicates (277 references) and retracted articles (*n* = 2) were subsequently removed. The remaining 548 articles were screened by titles and abstracts. During this stage of the review, 423 references were excluded for various reasons, including: incorrect sport type (e.g., basketball, rugby), wrong focus on SSGs (e.g., comparisons with other training methods), lack of analysis on the effect of relative pitch size, incorrect publication type (e.g., reviews), or examination of outcome variables beyond the scope of this review (e.g., decision-making index). After reading full texts (*n* = 125), a further 69 studies were excluded owing to the following reasons and/or combination of reasons: studies not performed in soccer, studies that did not compare two pitch sizes (or not with the same condition/rule), studies not reporting physical, physiological, technical, or tactical outcomes variables and/or the same were not identifiable through graphs. Therefore, 56 articles were eligible for this review (Figure 1). The included articles provided mean and SD for smaller and larger pitch sizes data for the mentioned outcome variables. An overview of the included studies can be found in Supplementary Table S3.

### 3.2 Risk of bias assessment

**Figure 2:**
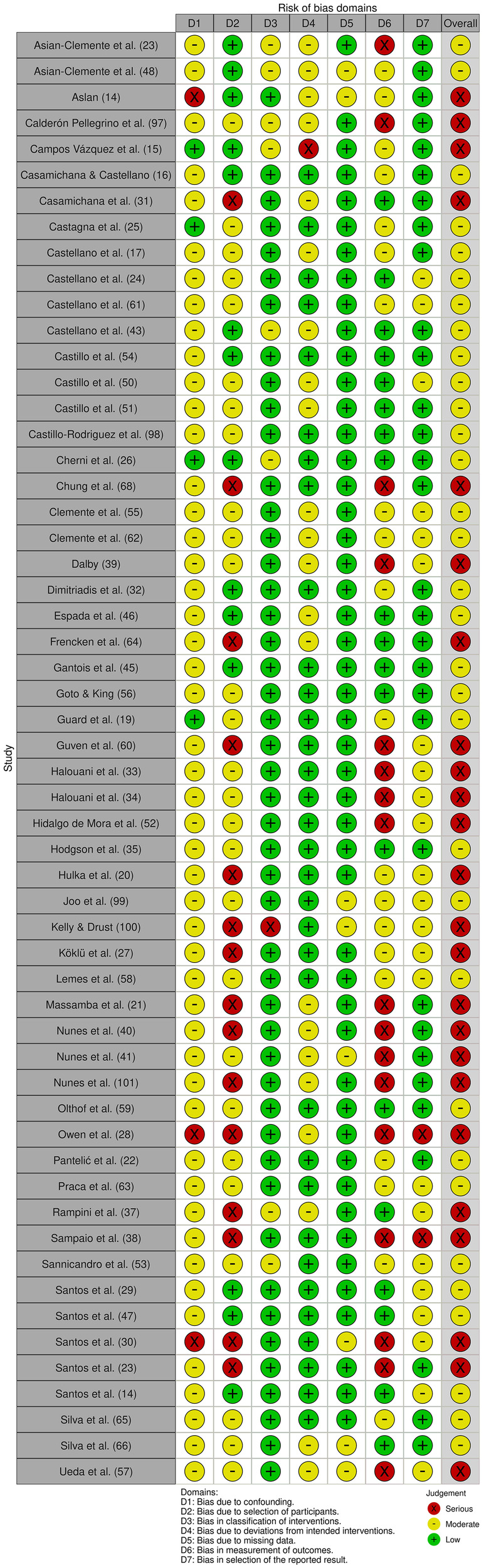
Traffic light plot of the risk of bias assessment.

**Figure 3:**
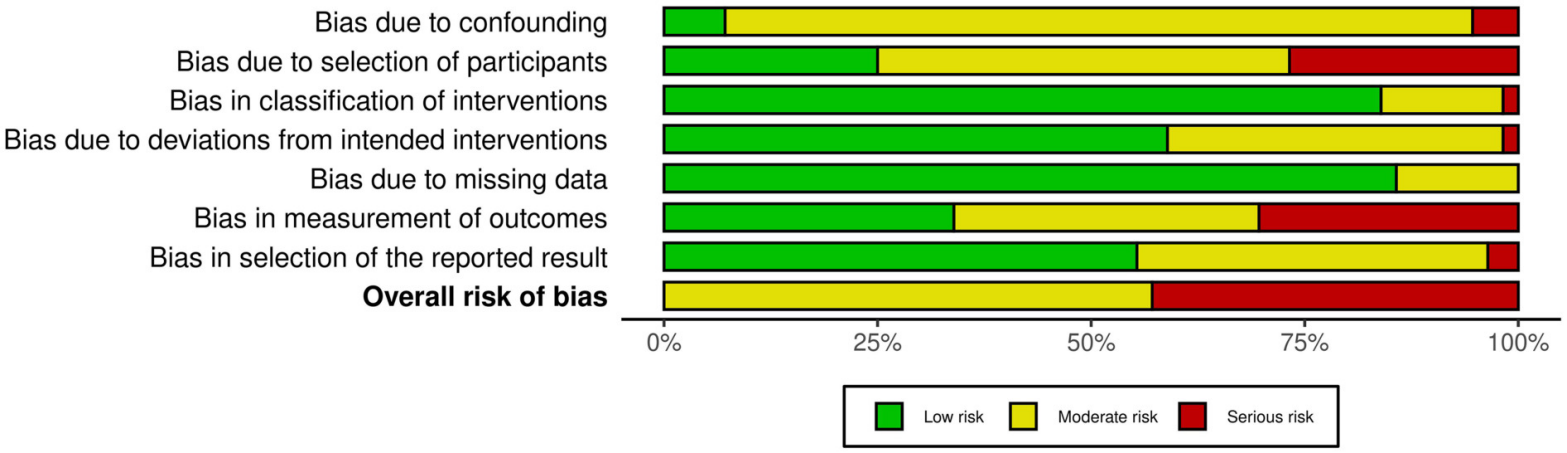
Summary plot of the risk of bias assessment.

A summary of the risk of bias assessment is provided in Figures 2, 3. Of the 56 included studies, 32 were rated as having moderate concerns, primarily due to inadequate control of potential confounders and limited reporting on participant selection criteria. 24 studies were assessed as having serious concerns, mainly due to outcome measurement bias (e.g., lack of blinding, insufficient methodological detail) and the absence of reported eligibility criteria for participant selection.

### 3.3 Physiological variables

A total of 35 studies (Supplementary Table S3) investigated the physiological responses associated with variations in relative pitch size. Of these, 11 studies examined HRmax > 85% (12, 14, 16, 17, 20, 27, 38, 44, 49, 55, 63), 4 studies HRmax 75%–85% (14, 20, 27, 44), 7 studies HRmax < 75% (14, 16, 17, 21, 27, 44, 63), 13 studies HRmax (12, 14, 17, 19, 20, 27, 28, 47, 54, 55, 60, 62, 63), 23 studies HRmean (14, 17–20, 27, 28, 33, 38, 40, 41, 43, 44, 46, 47, 49, 54, 55, 57, 58, 60, 62, 63), 18 studies RPE (12, 14, 17–19, 28, 32, 33, 38, 40, 41, 44, 47, 50–52, 57, 58), 10 studies Player Load (18, 20, 21, 23, 36, 38, 55, 62–64), 5 studies lactate (19, 28, 40, 41, 57), and 3 studies Edward's TRIMP (34, 38, 61). Lactate (%change and total change in mean) and RPE (%change and total change in mean) were significantly (*p* < 0.05) affected by relative pitch size. The results for all physiological variables can be observed in Table 2.

**Table 2:** Regression analyses for physiological variables.

Variable	N	Df	F	*P*-level	*R* ^2^	Equation
HRmean %change	150	1	0.21	0.65		
HRmean TM-change	150	1	3.58	0.06		
HRmax %change	72	1	0.50	0.48		
HRmax TM-change	72	1	0.05	0.82		
HRmax >85% %change	47	1	0.39	0.54		
HRmax >85% TM-change	47	1	0.02	0.90		
HRmax 75–85% %change	14	1	0.54	0.48		
HRmax75–85% TM-change	14	1	0.68	0.43		
HRmax <75% %change	32	1	0.17	0.68		
HRmax <75% TM-change	32	1	0.42	0.52		
Lactate %change*	45	1	9.35	0.01	0.18	0.16 x—4.08
Lactate TM-change*	45	1	70.86	<0.001	0.61	0.03 x—0.85
Edwards’ TRIMP %change	15	1	4.24	0.04	0.25	0.7 x—0.73
Edwards’ TRIMP TM-change	15	1	0.10	0.76		
Player Load %change	66	1	0.34	0.56		
Player Load TM-change	66	1	0.000	0.88		
RPE %change*	116	1	18.13	<0.001	0.14	0.11 x + 2.1
RPE TM-change*	116	1	9.2	<0.05	0.075	0.002 x + 0.46

* *p* < 0.05.

HR, heart rate; TM-change, change in total mean; RPE, rating of perceived exertion; TRIMP, training impulse.

### 3.4 Physical variables

A total of 37 studies examined the effect of relative pitch size on physical variables. Of these, 18 studies examined accelerations (12, 13, 15, 18, 19, 25, 26, 28, 33, 34, 36, 38, 42, 43, 59, 60, 63, 64), 18 studies decelerations (12, 13, 15, 18, 19, 25, 26, 28, 33, 34, 36, 38, 42, 43, 59, 60, 63, 64), 29 studies total distance (12, 13, 15, 16, 19–21, 23–25, 27, 28, 30, 32–34, 36–38, 42–45, 48, 53, 60, 61, 63, 64), 7 studies relative total distance (16, 18, 27, 32, 33, 36, 38), 21 studies sprinting distance (13, 15, 20, 21, 24, 27, 30, 32–34, 38, 42, 43, 45, 50–53, 55, 61, 64), 21 studies max. speed (13, 15, 16, 18, 21, 23, 24, 26–28, 32, 36, 38, 42, 50–52, 55, 59, 60, 63) and jogging distance (15, 17, 20, 21, 23, 25–28, 30, 32–34, 36, 38, 45, 48, 50, 52, 59, 61), 11 studies high-speed running distance (15, 23, 27, 28, 34, 36, 43, 45, 53, 59, 61), 24 studies running distance (15, 17, 20, 21, 23–25, 27, 28, 30, 32–34, 36, 37, 42, 45, 48, 51, 55, 59–61, 63), 20 studies walking distance (15, 17, 20, 21, 23, 25, 27, 28, 30, 32–34, 42, 45, 48, 50–52, 59, 61), 5 studies work-to-rest ratio (16, 20, 21, 23, 38), 1 study change of direction (18), 7 studies high-metabolic load distance (19, 34, 36, 37, 59, 61, 64), and 5 studies the number of sprints (25, 26, 30, 51, 55). Total and relative distance covered, maximal speed, work-to-rest ratio, high-metabolic load distance (% change and change in total mean), sprinting distance (% change), high-speed running distance (change in total mean), running distance (change in total mean), jogging distance (% change and change in total mean), walking distance (% change), as well as acceleration and deceleration (change in total mean) were significantly influenced by relative pitch size. A comprehensive overview of all physical variables is provided in Table 3.

**Table 3:** Regression analyses for physical variables.

Variable	N	Df	F	*P*-level	*R* ^2^	Equation
Total distance %change*	137	1	59.5	<0.001	0.31	0.06 x + 7.37
Total distance TM-change*	137	1	12.48	<0.001	0.09	0.34 x + 94.29
Relative total distance %change*	22	1	40.01	<0.001	0.67	0.12 x + 6.59
Relative total distance TM-change*	22	1	25.98	<0.001	0.57	0.09 x + 8.28
Max. speed %change*	112	1	48.40	<0.001	0.31	0.061 x + 3.9
Max. speed TM-change*	112	1	12.63	<0.001	0.10	0.01 x + 0.9
Number of sprints %change*	18	1	27.22	<0.001	0.63	3.56 x—112.69
Number of sprints TM-change	18	1	0.12	0.16		
Sprinting distance %change	138	1	2.71	0.10		
Sprinting distance TM-change*	138	1	61.75	<0.001	0.31	0.35 x —5.99
High-speed running distance %change	66	1	4	0.051		
High-speed running distance TM-change*	66	1	18.31	<0.001	0.22	0.12 x + 5.38
Running distance %change	143	1	0.000	0.97		
Running distance TM-change*	143	1	2.95	<0.001	0.26	0.49 x + 7.12
Jogging distance %change*	122	1	23.06	<0.001	0.16	0.16 x + 8.74
Jogging distance TM-change*	122	1	8.78	0.004	0.07	1.19 x—56.85
Walking distance %change*	133	1	11.08	<0.001	0.1	−0.05 x—2.93
Walking distance TM-change	133	1	3.63	0.1		
Acceleration %change	66	1	0.27	0.61		
Acceleration TM-change*	66	1	13.25	<0.001	0.17	0.28 x—6.17
Deceleration %change	65	1	0.19	0.66		
Deceleration TM-change	65	1	14.15	<0.001	0.18	0.26 x—4.57
Distance Individual Speed %change	78	1	0.1	0.75		
Distance Individual Speed TM-change	78	1	0.11	0.74		
HMLD %change*	15	1	13.20	0.003	0.50	0.31 x + 5.13
HMLD TM-change*	15	1	17.91	<0.001	0.58	1.58 x—43.95
HMLT Time %change	6	1	0.04	0.85		
HMLT TM-change	6	1	0.69	0.45		
COD %change	8	1	0.03	0.67		
COD TM-change	8	1	1.27	0.30		
Work-to-Rest ratio %change*	35	1	27.46	<0.001	0.45	0.39 x + 15.94
Work-to-Rest TM-change*	35	1	0.02	0.47		

* *p* < 0.05.

TM-change, change in total mean; TD, total distance; HMLD, high-metabolic load distance; HMLT, high-metabolic load time; COD, change of direction.

### 3.5 Technical variables

A total of 12 studies were identified that investigated the effect of relative pitch size on technical variables. Of these, 5 studies examined ball possessions (14, 28, 39, 49, 53), 5 studies dribbles (14, 16, 39, 43, 49), 10 studies passes (14, 28, 39, 43, 45, 46, 49–52), 6 studies turnovers (14, 16, 28, 39, 43, 46), 7 studies shots (14, 16, 39, 43, 45, 46, 53), and 2 studies ball touches (45, 49). Ball touches (%change and change in total mean) and ball possession (%change and change in total mean), dribbles (%change) and turnover (%change and change in total mean) were significantly affected by relative pitch size. The results for all technical variables can be observed in Table 4.

**Table 4:** Regression analyses for technical variables.

Variable	N	Df	F	*P*-level	*R* ^2^	Equation
Ball touches %change*	220	1	25.55	<0.001	0.11	−0.33 x + 27.59
Ball touches TM-change*	220	1	7.36	0.01	0.03	−0.04 x + 2.51
Ball possession %change*	15	1	4.63	0.01	0.26	0.65 x—69.46
Ball possession TM-change*	15	1	6.98	0.02	0.35	0.03 x—2.77
Passes %change	57	1	0.21	0.65		
Passes TM-change	57	1	0.01	0.92		
Dribbles %change*	15	1	11.14	0.01	0.46	−0.89 x + 86.22
Dribbles TM-change	15	1	1.1	0.31		
Shots %change	17	1	1.1	0.31		
Shots TM-change	17	1	0.65	0.43		
Turnover %change*	29	1	37.58	<0.001	0.58	−0.27 x + 9.6
Turnover TM-change*	29	1	67.41	<0.001	0.71	−0.04 x + 1.41

* *p* < 0.05.

TM-change, change in total mean.

### 3.6 Tactical variables

A total of 10 studies investigated changes in tactical variables resulting from variations in relative pitch size. Of these, 4 studies examined stretch index (22, 31, 53, 56), inter-team distance (22, 35, 53, 65), spatial exploration index (30, 56, 66, 67), and surface area (35, 53, 65, 66). 2 studies examined team width (22, 29) and studies team length (22, 29), while 3 studies examined the length per width ratio (53, 56, 65). Team width (TM-change), surface area (%change), inter-team distance (%change), stretch-index (%change and total change in mean) and spatial exploration index (%change), were significantly affected by relative pitch size. The results for all tactical variables can be observed in Table 5.

**Table 5:** Regression analyses for tactical variables.

Variable	N	Df	F	*P*-level	R^2^	Equation
Team width %change	20	1	4.31	0.05		
Team width TM-change*	20	1	15.55	<0.001	0.46	−0.19 x + 32.36
Team length %change	20	1	0.11	0.75		
Team length TM-change	20	1	1.61	0.22		
Surface area %change*	19	1	5.25	0.04	0.24	0.31 x + 11.79
Surface area TM-change	19	1	4.21	0.06		
Stretch index %change*	17	1	7.39	0.02	0.33	0.22 x + 6.85
Stretch index TM-change*	17	1	32.89	<0.001	0.69	0.08 x—1.17
Inter-team distance %change*	30	1	40.47	<0.001	0.59	1.66 x—44.57
Inter-team distance TM-change	30	1	2.58	0.12		
Length per width ratio %change	15	1	0.11	0.75		
Length per width ratio TM-change	15	1	0.15	0.71		
Spatial exploration index %change*	11	1	55.82	<0.001	0.86	0.36 x—6.98
Spatial exploration index TM-change	11	1	252	0.89		

* *p* < 0.05.

TM-change, change in total mean.

### 3.7 Changes in variables due to relative pitch size

Tables 6–9 displays different percentages increase in the relative pitch size and its effect on the significant physiological, physical, technical and tactical variables respectively, based on the individual variables linear regression equations provided in Tables 2–5.

**Table 6:** Physiological variables change (% or total) with % relative pitch size increase.

Variable	10%	20%	30%	50%	75%	100%	150%
Lactate %change			0.74	3.96	8	12.01	20.06
Lactate TM-change				0.55	1.25	1.95	3.35
Edwards’ TRIMP %change	−0.03	1.33	2.03	3.43	5.18	6.93	10.4
RPE %change	3.18	4.26	5.34	7.5	10.2	12.9	18.3
RPE TM-change	0.48	0.5	0.52	0.56	0.61	0.66	0.76

TM-change, change in total mean; RPE, rating of perceived exertion; TRIMP, training impulse.

**Table 7:** Physical variables change (% or total) with % relative pitch size increase.

Variable	10%	20%	30%	50%	75%	100%	150%
TD %change	7.97	8.57	9.17	10.37	11.87	13.37	16.37
TD TM-change	97.69	101.09	104.49	111.29	119.79	128.29	145.29
Relative TD %change	7.77	8.95	10.1	12.5	15.4	18.4	24.3
Relative TD TM-change	9.20	10.1	11.0	12.9	15.2	17.5	22.1
Max. Speed %change	4.51	5.12	5.73	6.95	8.47	10	13.05
Max. Speed TM-change	0.99	1.1	1.2	1.3	1.65	1.89	2.39
Number of sprints %change				65.3	154	243	421
Sprinting distance TM-change		0.93	4.39	11.31	20	28.61	45.91
High-speed running distance TM-change	6.57	7.76	8.95	11.33	14.31	17.28	23.23
Jogging distance %change	10.37	12	13.63	16.9	21	25.04	33.19
Jogging distance TM-change				2.61	32.34	62.1	121.5
Walking distance %change	−3.42	−3.89	−4.36	−5.30	−6.48	−7.65	−10
Running distance TM-change	12.02	16.92	21.82	31.62	43.87	56.12	80.62
Acceleration TM-change			2.26	7.94	14.99	22.04	36.14
Deceleration TM-change		0.6	3.18	8.34	14.79	21.24	34.14
HMLD %change	8.26	11.4	14.5	20.8	28.6	36.4	52.1
HMLD TM-change			3.54	35.2	74.8	114	193
Work-to-Rest ratio %change	19.9	23.8	27.8	35.6	45.5	55.3	75.0

TM-change, change in total mean; TD, total distance; HMLD, high-metabolic load distance; HMLT, high-metabolic load time. Negative values indicate a relative decrease compared to the intercept of the regression equation, which serves as the baseline. These values do not represent an actual negative outcome but rather illustrate a decline in the variable as RPS increases.

**Table 8:** Technical variables change (% or total) with % relative pitch size increase.

Variable	10%	20%	30%	50%	75%	100%	150%
Ball touches %change	24.3	21.0	17.8	11.2	2.99	−5.21	−21.6
Ball touches TM-change	2.11	1.71	1.31	0.51	−0.49	−1.49	−3.49
Ball possession %change	−62.9	−56.4	−49.9	−36.9	−20.6	−4.36	28.2
Ball possession TM-change	−2.52	−2.27	−2.02	−1.52	−0.9	−0.27	0.98
Dribbles %change	77.3	68.3	59.4	41.6	19.2	−3.09	−47.7
Turnover %change	6.92	4.25	1.58	−3.76	−10.43	−17.11	−30.46
Turnover TM-change	1.01	0.61	0.21	−0.59	−1.59	−2.59	−4.6

TM-change, change in total mean. Negative values indicate a relative decrease compared to the intercept of the regression equation, which serves as the baseline. These values do not represent an actual negative outcome but rather illustrate a decline in the variable as RPS increases.

**Table 9:** Tactical variables change (% or total) with % relative pitch size increase.

Variable	10%	20%	30%	50%	75%	100%	150%
Team width TM-change	30.5	28.6	26.8	23.1	18.4	13.8	4.46
Surface area %change	14.8	17.9	21.0	27.1	34.7	42.4	57.7
Surface area TM-change	19.4	22.7	26.0	32.7	41.0	49.3	65.9
Stretch index %change	9.08	11.3	13.5	18.0	23.6	29.2	40.3
Stretch index TM-change	1.92	2.67	3.42	4.92	6.79	8.67	12.4
Inter-team distance %change	61.1	77.7	94.2	127	169	210	292
Spatial exploration index %change	3.39	1.01	−1.38	−6.14	−12.1	−18.0	−29.9
Spatial exploration index TM-change	0.99	−0.92	−2.83	−6.65	−11.4	−16.2	−25.8

TM-change, change in total mean. Negative values indicate a relative decrease compared to the intercept of the regression equation, which serves as the baseline. These values do not represent an actual negative outcome but rather illustrate a decline in the variable as RPS increases.

Table 6 provides an overview of the observed increases in physiological parameters relative to changes in relative pitch size. Specificially, RPE (%change and absolute change in total mean) increased with increasing relative pitch size. Similarly, blood lactate levels (%change and absolute change in total mean) also rose with larger relative pitch sizes; however, a positive effect was only observed after approximately a 30% increase. A comparable trend was noted for Edwards’ TRIMP, with significant changes occurring after a 10% increase in relative pitch size (Table 6).

Twelve out of sixteen physical variables demonstrated a significant increase with a relative pitch size increase of more than 10%, including total distance, relative total distance, and maximal speed, all in terms of both percentage change and absolute total mean change (Table 7). Additionally, sprinting distance, high-speed running distance (absolute mean change), running distance, jogging distance, high-metabolic load distance (HMLD), and work-to-rest ratio (%change) also increased under these conditions. In contrast, walking distance (%change) decreased with a relative pitch size increase of more than 10%. Acceleration and deceleration (absolute mean change) demonstrated a positive response, with acceleration increasing in response to a 30% increase in relative pitch size and deceleration showing a positive effect with a 20% increase. However, the effects on HMLD (absolute mean change) and the number of sprints (%change) were delayed, with significant changes occurring only after a 28% and 30% increase in relative pitch size, respectively. Table 7 presents the observed increases in physical variables in relation to practical percentage increases in relative pitch size.

The results of the regression equation for technical variables are presented in Table 8. All technical variables except for ball possession decreased with increasing percentage relative pitch size. More specifically, turnover (%change) showed negative values starting from ∼25% increase in relative pitch size, ball touches (change in total mean) from ∼65%, turnovers (change in total mean) from ∼80%, ball touches (%change) from ∼85%, dribbles (%change) from ∼95%, ball possession (%change) from ∼105% and ball possession (change in total mean) from ∼105% increase in relative total pitch size.

Table 9 displays changes in tactical variables in relation to practical percentage increases in relative pitch size. The following tactical variables increased with increasing pitch size, i.e., surface area, inter-team distance and stretch-index while team width and spatial exploration index decreased with increase in relative pitch size.

## 4 Discussion

The primary purpose was to quantify changes in physiological, physical, technical and tactical outcome variables due to changes in relative pitch size and to give practical guides with regards to each of the mentioned category. It can be concluded that relative pitch size might have effect on physiological (i.e., Lactate, Edwards' TRIMP and RPE) variables. The increase in Lactate, Edwards' TRIMP and RPE (Table 6) in this investigation is in line with previous scientific research (1, 4, 6). However, the results of this investigation reflect the actual changes in relative pitch size by accounting for the relationship between the number of players and pitch size in conjunction with the aforementioned physiological variables. From a practical point of view an approximate increase of 30% in relative pitch size will not necessarily impact Lactate. However, a 50%, 100% and 150% increase in relative pitch size resulted in ∼4, ∼12 and ∼20% increase in Lactate (Table 6). In total terms, the same percentage increase, resulted in increase in Lactate of 0.55, 1.95 and 3.35 mmol/L. If a practitioner uses Edwards' TRIMP to measure exercise intensity, a 50%, 100%, and 150% increase in relative pitch size results in an approximate increase of ∼3%, ∼7%, and ∼10% in arbitrary units (AU), respectively (Table 6). Coaches can expect a percentage increase in RPE of ∼5, ∼10 and ∼18 with an increase of 30%, 75%, and 150% in percentage of relative pitch size, respectively (Table 6). While the aforementioned variables have been widely used to assess intensity in football, scientists and coaches should critically evaluate their validity. Specifically, intensity has been discussed in relation to internal load, which is often quantified using these variables and subsequently correlated with HR measures. However, the relationship between HR and the intermittent nature of football remains problematic (69). More precisely, scientific literature suggests that HR tends to underestimate the intensity of smaller drills (70). Furthermore, HR has been reported to demonstrate relatively low inter-subject variability (1.3%–2.2%), leading to concerns regarding its acceptability as a reliable intensity marker (70). Moreover, it seems that HR alone is unable to reflect task intensity in football due to the intermittent nature (71, 72) as well as the different type of practices including heavy neuromuscular components (71). Consequently, it seemed not surprising that HR measures were not affected by an increase in relative pitch size, however, still opposing the limited scientific literature and indicating a multifaceted relationship between HR and so-called task constraints during SSGs (4, 5). When player numbers were kept constant, a larger playing area increased the intensity (3) and therefore the HR of players in the SSGs (4). However, it was also mentioned that smaller SSGs, i.e., 3 vs. 3 compared to 5 vs. 5, resulted in higher HRs (3). Consequently, it was suggested to evaluate exercise intensity in relation to reserve HR due to its proposed greater reliability as an indicator of HR (69). Nevertheless, time spent in HR zones (i.e., >80% HRmax) was also not significantly different with increasing relative pitch size. HR also differed from RPE significantly over different SSGs (70) and it was suggested that with regards to subjective perception of exercise intensity RPE may depict a better representation of the internal load (70) also having acceptable inter-subject variability of 5.1%–9.9% over a wide range of soccer training (70). Consequently, RPE was seen as a useful parameter of internal load during non-continuous (e.g., intermittent and sprint) exercises (73). Edwards's TRIMP correlated with average HR (0.33, *p* < 0.01) (74) and displayed good convergent validity (75). However, from a training perspective it was also suggested as a limited load monitoring tools to indicate the reality of training situations (high-intensity action with recovery intervals) (74). With regards to Lactate, it was suggested that the majority of the reported results during SSGs with a smaller number of players statistically increase the lactate concentration (4). Technically, comparing that statement directly with the results of this study seems insufficient, as multiple scientific sources were collated without consistently considering relative pitch size alongside the decrease in the number of players. Therefore, further research is needed to examine physiological variables during SSGs while explicitly accounting for relative pitch size.

The majority of variables investigated where physical in nature and twelve variables increased with larger relative pitch size. In accordance with present evidence (5, 6), total distance, high-speed running distance and jogging distance were observed to increase with relative pitch size. It is reasonable to assume that greater relative pitch space allows players to cover longer distances and attain higher running velocities (5). Consequently, the maximum speed at which the players operate as well as the sprinting distance increased with greater relative pitch size in this investigation. Specifically, %changes in total distance with a 50% and 100% increase in relative pitch size were approximately 10% and 13%, respectively, with total distances recorded at approximately 111 meters and 128 meters (Table 7). Considering these values with regards to a training perspective, an increase of over 10% (i.e., 11% and more) in total distance running was controversially debated throughout the literature (76), however also showing that the equivalent increase in training load might already result in injury consequences when chronic workloads are also high calling for the so-called “ceiling of safety” (76). High-speed running distance changed by approximately 11 and 17 m with a 50% and 100% increase in relative pitch size, respectively (Table 7). The moderating effect of relative pitch size on high-speed running distance is reflected in the study by Dello Iacono et al. (9), who found an increase of 2.5 m/min, 2.8 m/min, and 1.9 m/min for small-sided games (2 vs. 2 to 4 vs. 4), medium-sided games (5 vs. 5 to 7 vs. 7), and large-sided games (8 vs. 8 to 10 vs. 10), respectively. Coaches might achieve an approximate increase of ∼7 and ∼10% of maximum speed with an increase of 50% and 100% in relative pitch size. On the other hand, the threshold effect observed in specific high-intensity variables (<30% relative pitch size in maximum speed, and high-speed running) should be considered. A similar pattern has been reported by Sangnier et al. (10), demonstrating that a reduced relative pitch size (<50 m² per player) is associated with decreased sprint distance and fewer sprint efforts. This suggests that smaller playing areas may not provide sufficient space to stimulate speed-oriented actions during SSGs (17). Furthermore, Sangnier et al. (10) observed that acceleration and deceleration distances per minute follow a logarithmic trend, with notably lower values recorded as relative pitch size decreases. Although the authors did not explicitly analyze percentage reductions in pitch size, a comparable decline in accelerative and decelerative activities was observed below a threshold of 150 m^2^ per player, approximately 42% of the highest reported value (350 m^2^ per player). These observations align partly with our findings, which indicate that the frequency of acceleration and deceleration events tends to plateau following an approximate 30% reduction in relative pitch size (Table 7). In contrast, recent meta-analyses (6), have reported similar acceleration and deceleration frequencies across both small and large pitch configurations, thereby highlighting the potential significance of relative pitch size as a moderating factor in players' physical output. Overall, the scaling of relative pitch size and the proposed thresholds presented in this review may offer valuable guidance for practitioners aiming to optimize training protocols, particularly for enhancing anaerobic performance capacities.

Since HMLD represents the summation of all actions exceeding 25.5 W/kg (77) and considering that power is defined as work over time, it is reasonable to expect an increase in HMLD with increasing relative pitch size. This effect is particularly pronounced when more space per player allows for extended periods at high speeds. Specifically, an increase of approximately 36% in HMLD appears achievable with a 100% increase in relative pitch size. However, HMLD has shown poor reliability values when derived from global positioning systems for intensities exceeding 20 W/kg, which raises concerns about its utility for monitoring purposes in soccer (78). The work-to-rest ratio was positively affected by an increase in relative pitch size. Specifically, a 10%, 20%, and 50% increase in relative pitch size led to approximately 20%, 24%, and 36% increases in the work-to-rest ratio, respectively (Table 7). Considering the increases in physical parameters associated with larger relative pitch sizes, while maintaining consistent protocols (e.g., bout duration), a higher work-to-rest ratio can be anticipated as a logical consequence. The work-to-rest ratio has received considerable attention in the literature due to its importance in soccer (79, 80). For instance, most high-intensity actions seem to occur after recovery durations of over 60 s (80). However, position-specific variations were observed, with midfielders exhibiting shorter recovery times, often less than 20 s for central midfielders (80, 81). Consequently, the work-to-rest ratio between high-intensity bouts increased from 1:12 for the match average to 1:2 during the most intense period in professional English Premier League Football (79). Therefore, improving the work-to-rest ratio, as well as other related variables, appears to be desirable. Indeed, it has been suggested that prescribing work intervals of a few minutes at intensities exceeding 90% HRmax (i.e., aerobic high-intensity training) may lead to beneficial adaptations in aerobic power and capacity (82). However, when work intervals are conducted at much higher intensities, as all-out efforts or sprinting of typically 10- to 40 s duration with longer recovery periods (i.e., speed endurance training), beneficial adaptations pertaining to anaerobic energy systems, ion handling, and fatigue resilience were observed (82). Consequently, altering the work-to-rest ratio with increasing relative pitch size in SSGs might benefit physical ability in soccer players.

Technical parameters also changed with increase in relative pitch size. That is, less ball touches, dribbles and turnovers, but more possession was observed with increasing relative pitch size (Table 8). Ball touches were measured based on (non-)dominant foot contacts, ball touches per possession, number of receptions, and the percentage of ball touches. These values were used to represent each player's involvement in the game. In detail, an increase of 10, 20, and 30% in relative pitch size resulted in a decrease of approximately 63%, 56%, and 50% in ball touches, respectively (Table 8). Therefore, it can be concluded that an increase in relative pitch size may result in a decrease in the individual technical involvement of players. Indeed, the reduction in the number of players in the relative pitch size increased the total ball touches per player and, therefore, the number of technical actions (7). Similarly in this investigation, the increase in relative pitch size showed a decrease in percent ball touches. Adding to this notion, dribbling as another key parameter in football (83) was also observed to decrease with increasing pitch size. More specifically, a steady decrease in dribbles was observed with an increase in relative pitch size. This finding adds to the existing, albeit conflicting, body of research, which indicates a reduction in dribbling with increasing pitch size (3) while other studies suggest no significant effect (6). As sport-specific technical skills are a central component in the development of young athletes (84) and in combination with the observation that technical ability was a crucial factor in career progression (85) it seems reasonable to conclude that the utilization of great(er) relative pitch size might affect player's skill development negatively. Furthermore, as research increasingly supports the use of SSGs to replicate the dynamic, unpredictable nature of competitive matches (86, 87), it is essential to recognize the potential effects of specific task constraints, such as relative pitch size, on the frequency of technical execution. Moreover, following stated principles of non-linear pedagogy (87), Chow et al. (88) proposed that task constraints should mirror the formal game context to facilitate effective learning. In this context, including exploratory learning and the targeted manipulation of constraints (e.g., reduced relative pitch size) could encourage individualized problem-solving to meet specific game demands (89), fostering a higher density of technical actions (90). Based on the results of our study, however, these learning principles may not be fully achieved in SSG formats with larger relative pitch sizes. Therefore, to replicate training in an integrative and representative manner, a reduced relative pitch size could serve as a valuable task constraint for enhancing technical density, particularly in youth soccer players. However, variations in expertise levels must be considered, as task constraints may limit the success of sport-specific actions (87). Further research is needed to explore how adjustments in relative pitch size could benefit players at different levels of expertise in soccer.

Ultimately, there was a decrease in turnovers observed with an increase in relative pitch size. A possible explanation might be the greater availability of individual space and therefore time resulting in less initial opponent pressure (91) for better decision making and technical execution concluding in possibly less turnovers. In this study a 10%, 20% and 30% increase in relative pitch size resulted in approx. ∼7%, ∼4% and ∼2% turnovers, respectively (Table 8). With regards to soccer practice, players with lower-level technical proficiency might benefit from greater relative pitch size for better football performance. With a decrease in turnovers, it would seem reasonable to expect ball possession increase with greater relative pitch size. Indeed, ball possession increased with each 10% increase in relative pitch size.

Regarding tactical variables, it can be summarized that surface area, stretch index, and inter-team distance increased, while team width and spatial exploration index decreased with an increase in relative pitch size. Consequently, the initial two variables seem to be prone to increase purely on space availability in both axis (width and length) (6), which also appeared in this investigation. More precisely, the stretch index increased by ∼9%, ∼11%, and ∼14% with a 10%, 20%, and 30% increase in relative pitch size, respectively. Similarly, surface area increased by ∼15%, ∼18%, and 21% with a 10%, 20%, and 30% increase in relative pitch size, respectively (Table 9). Therefore, the results of this research align with previous investigations (6). However, as it was also suggested that increasing a specific axis of the field (i.e., length) players tend to expand more accordingly to this axis (92) therefore leaving some necessity for further research in this area clarifying the effect of manipulating one or two field dimension and its effect on tactical variables. As team width decreased by ∼1.6% for every 10% increase in relative pitch size (Table 9), it can be speculated from this review that the increase in relative space per player does not necessarily lead to a proportional expansion of utilized space in all directions. Instead, the adaptation appears to be primarily reflected in an increase in team length rather than width, suggesting that teams tend to stretch more vertically rather than laterally as pitch size increases (65). As expected, with an increase in pitch size, players are anticipated to be positioned farther apart from one another (6). Consequently, the observed increase of approximately 60%, 77%, and 95% in inter-team distance with a 10%, 20%, and 30% increase in relative pitch size aligns with existing scientific knowledge. However, it is important to note that the same authors did not find a difference in centroid position with increasing pitch size (6). It was also suggested that the goal-to-goal centroid difference is more influenced by changes in pitch size than the lateral axis distance, further confirming both previous findings (6).

The spatial exploration index defined as the average difference between a player's average positions and their actual position at each moment of the game (93), suggests that an increase in individual relative pitch size allows for greater spatial exploration (94). However, the variable decreases with increasing relative pitch size, in percentage as well as total value. In light of these findings, the perception of environmental properties as affordances (i.e., opportunities for action) (86) might provide a theoretical rationale for tactical adaptations resulting from spatial constraints, such as changes in relative pitch size. From an ecological dynamics perspective, increasing relative pitch size may promote the emergence of more structured positional behaviors, as players become attuned to role-specific affordances shaped by the broader spatial configuration and inherent task demands, such as maintaining greater positional stability to preserve the team's structural organization (93). Moreover, larger relative pitch sizes may attenuate opportunities to disrupt the opposition's structural organization, as increased spatial distribution can reinforce team integrity and reduce the likelihood of provoking tactical instability (30, 95). These reduced interpersonal coordination demands and diminished immediate pressure from opponents, common in larger relative pitch sizes, may limit the emergence of co-adaptive behaviors and mutual attunement among players (96). Consequently, this could result in lower levels of spatial exploration and adaptability. Furthermore, externally imposed coaching constraints (e.g., rigid tactical instructions or role assignments) may act as informational filters, attenuating the perception and utilization of available affordances (88). Thus, while increased relative pitch size theoretically offers a greater range of movement possibilities, the actual exploitation of these affordances may be constrained by both environmental properties and imposed task structures. Given the mixed findings in current research, further investigation is warranted to clarify how spatial manipulations interact with tactical performance in soccer.

## 5 Practical implications

The present findings provide practical insights for practitioners aiming to manipulate relative pitch size in SSGs for targeted training outcomes (Tables 6–9). Smaller pitch sizes enhance technical involvement while reducing intensity, making them valuable for recovery or low-intensity skill sessions. These formats may facilitate the development of tactical awareness and technical precision without overwhelming players, particularly during post-match or off-day recovery. Conversely, larger pitch sizes increase physical demands (e.g., total distance, high-speed running) and physiological load (e.g., elevated lactate concentrations and RPE), which are beneficial for overload training aimed at enhancing endurance and stimulating high-intensity actions, typically incorporated during peak or intensity-focused phases of the training cycle. The manipulation of relative pitch size can also support periodization strategies, particularly in pre-season phases, by progressively adapting players to larger relative pitch sizes, thus mitigating the risk of early overreaching in high-intensity parameters. This adaptation helps to prevent potential injury resulting from an extended mismatch between acute and chronic training loads. For youth players with lower training age, smaller relative pitch sizes offer a promising training regimen for enhancing technical skills, decision-making, and tactical awareness within constrained spaces under game-like situations in SSGs. As players mature, it is essential to adjust relative pitch sizes to simulate the physical demands of competitive matches, especially during the transition from junior to senior soccer. Greater relative pitch size fosters position-specific adaptations that are important in player profile development. More specifically, positions with high physical game demands, such as wingers and full-backs, will benefit from larger pitch sizes to improve sprinting capabilities and high-intensity actions. Finally, careful attention must be given to balancing work-to-rest ratios, particularly for youth players in growth phases, where the risk of overtraining seems to be heightened. Integrating players undergoing rapid growth as floaters in SSGs with larger relative pitch sizes may be an effective strategy to manage training load while supporting physical development. This allows for moderated demands and continued gameplay involvement without imposing excessive mechanical or physiological strain during sensitive maturation phases.

## 6 Limitations

This study has several limitations. First, the way variables were grouped presents a potential issue. For instance, the grouping of percentages of HRmax and speed zones, due to the variability of thresholds across the scientific literature, may not accurately reflect actual values. Additionally, some variables consist of multiple sub-parameters that follow a logical categorization but were not explicitly defined as the primary outcome variable in the study, which might again lead to an inaccurate summary of certain variables. Additionally, the percentage increase in relative pitch size was calculated solely based on the actual percentage difference between the investigated pitch sizes, without accounting for potential player dynamics from a numerical perspective. For instance, while a 3 vs. 3 and an 8 vs. 8 game format may share the same relative pitch size, they represent fundamentally different demands, which could influence the outcomes in distinct ways. Further, the effect of confounding factors, including task constraints (e.g., touch restrictions), participant characteristics (e.g., youth or adult players) or regimen modalities (e.g., playing and recovery times), was not accounted for. Although the linear regression model employed in this review provides statistical insights, the interaction between pitch size and potential confounding factors likely influences the outcomes in ways not fully captured by the model. To address this, multivariate models (e.g., multiple regression, analysis of covariance) should be employed in future research, with relevant covariates (e.g., age, expertise level) included to control for their potential effects on dependent variables. Moreover, due to the heterogeneity among the included studies, particularly in SSG characteristics and outcome measure reporting, caution is warranted when interpreting the validity and generalizability of the findings. While the broad scope of this review enables overarching conclusions, it currently limits the specificity of recommendations for distinct subgroups, such as different age categories, expertise levels, or genders. To enhance this, future studies should adopt narrower inclusion criteria (e.g., standardized SSG formats such as 4 vs. 4) and conduct subgroup or sensitivity analyses to account for contextual variability.

The predominance of cross-sectional study designs further restricts inferences regarding long-term or cumulative effects. While our regression models offer a preliminary understanding of acute responses to relative pitch size, longitudinal studies are needed to evaluate sustained training effects and to better inform periodization strategies. Lastly, variations in risk of bias across the included studies should be acknowledged. Many studies lacked methodological rigor, such as the absence of control groups, unclear participant selection processes, or insufficient control of confounding factors. Additionally, where feasible, the implementation of blinding procedures is also recommended to enhance internal validity. Such methodological advancements would facilitate a more accurate understanding of the effects of relative pitch size, thereby optimizing implementation strategies in soccer training contexts.

## 7 Conclusion

This review aimed to provide practical guidance on the effect of relative pitch size on physiological, physical, technical, and tactical parameters. The results revealed an association between alterations in relative pitch size and changes in outcome measures. Notwithstanding certain methodological limitations, the individual findings from the included studies suggest that increases in relative pitch size are associated with elevated physiological parameters (e.g., RPE), physical parameters (e.g., total distance), and tactical parameters (e.g., surface area), whereas technical measures (e.g., ball touches) tend to decrease as relative pitch size increases. However, other variables showed no meaningful association with changes in relative pitch size. It can be concluded that relative pitch size is a valuable constraint for the implementation of SSGs interventions, emphasizing physiological and physical demands while promoting technical proficiency and collective dynamics.
