# Supplementary material for: The effect of relative pitch size on physiological, physical, technical and tactical variables in small-sided games: a literature review and practical guide
Source: Front Sports Act Living. 2025 May 6;7:1592536. doi: 10.3389/fspor.2025.1592536 (PMC12089100; doi:10.3389/fspor.2025.1592536)
Supplement: Supplementary file 1 [file Table1.docx]

Supplementary Material

The effect of relative pitch size on physiological, physical, technical and tactical variables in small-sided games: A literature review and practical guide

Table S1: Search strategy for each database

| Web of Science Boolean Terms | **(ALL=(football)) OR ALL=(soccer) AND (ALL=(small sided*)) OR ALL=(conditioned game) AND**  **(ALL=(pitch)) OR ALL=(field)**  Filters used to narrow down returns included – Language: English, Portuguese; Document types: Article. 424 articles were returned. |
| --- | --- |
| PsycInfo Boolean Terms | (football OR soccer) AND (small-sided games OR conditioned games) AND (field OR pitch)  Filters used to narrow down returns included – Language: English, Portuguese; Population Group: Human; Publication type: All journals. 28 articles were returned. |
| PubMed Boolean Terms | ("Football"[MeSH Terms] OR "Soccer"[MeSH Terms]) AND ("small sided*"[All Fields] OR (("conditioning, psychological"[MeSH Terms] OR ("conditioning"[All Fields] AND "psychological"[All Fields]) OR "psychological conditioning"[All Fields] OR "conditioned"[All Fields] OR "conditioning"[All Fields] OR "conditionings"[All Fields]) AND ("game s"[All Fields] OR "games"[All Fields] OR "gaming"[All Fields]))) AND ("field"[All Fields] OR "field s"[All Fields] OR "fields"[All Fields] OR ("pitch"[All Fields] OR "pitched"[All Fields] OR "pitches"[All Fields] OR "pitching"[All Fields]))  No filters were applied. 123 articles were returned. |
| Scielo Boolean Terms | youth AND (soccer OR football) AND maturation AND bio-banding  Filters used to narrow down returns included – Language: English, article type: Review articles, Research articles, Bok chapters, Conference abstracts, Discussion, Other. 33 articles were returned. |
| SportDiscus Boolean Terms | (football OR soccer) AND (small-sided games OR conditioned games) AND (field OR pitch)  Filters used to narrow down returns included – Language: English, Publication type: Academic Journal. 224 articles were returned. |
