# Supplementary material for: The effect of relative pitch size on physiological, physical, technical and tactical variables in small-sided games: a literature review and practical guide
Source: Front Sports Act Living. 2025 May 6;7:1592536. doi: 10.3389/fspor.2025.1592536 (PMC12089100; doi:10.3389/fspor.2025.1592536)
Supplement: Supplementary file 2 [file Table2.docx]

Supplementary Material

The effect of relative pitch size on physiological, physical, technical and tactical variables in small-sided games: A literature review and practical guide

Tables S2: Assigned variables for each dimension

Physiological variables

| Variables in review | Variables in included studies |
| --- | --- |
| HRmean %change | %HRMean (%) (Casamichana & Castellano, 2010; Castellano et al., 2015), HRMean (bpm) (Aslan, 2013; Castagna et al., 2019; Castillo-Rodríguez et al., 2023; Cherni et al., 2025; Halouani et al., 2017a, 2017b; Hodgson et al., 2014; Kelly & Drust, 2009; Owen et al., 2004; Pantelić et al., 2019; Fernando Jorge Santos et al., 2021; Fernando J. Santos et al., 2021; F. J. Santos et al., 2021), %Hrmax (mean) (Casamichana et al., 2018; Dimitriadis et al., 2022; Guard et al., 2022; Massamba et al., 2020; Sampaio et al., 2007; Santos et al., 2022), HRaverage (bpm) (Hulka et al., 2016; Köklü et al., 2013; Rampinini et al., 2007) |
| HRmean TM-change |  |
| HRmax %change | HRMax (bpm) (Asian-Clemente et al., 2023; Aslan, 2013; Castagna et al., 2019; Castillo-Rodríguez et al., 2023; Owen et al., 2004; Santos et al., 2022; Fernando Jorge Santos et al., 2021; F. J. Santos et al., 2021), %HRMax (%) (Campos Vázquez et al., 2017; Casamichana & Castellano, 2010; Castellano et al., 2015; Köklü et al., 2013), Peak heart rate (bpm) (Pantelić et al., 2019), HRpeak (Cherni et al., 2025) |
| HRmax TM-change |  |
| HRmax >85% %change | HR >90% (s)(Asian-Clemente et al., 2023), >90% HRMax (Casamichana & Castellano, 2010), Percent playing time 84-90% HRMax (Castellano et al., 2015), Percent playing time >90% HRMax (Castellano et al., 2015; Guard et al., 2022; Massamba et al., 2020; Pantelić et al., 2019), HR 85-89% (Castillo-Rodríguez et al., 2023), HR >90% (Castillo-Rodríguez et al., 2023), Heart rate >85% Max (Campos Vázquez et al., 2017; Hulka et al., 2016; Massamba et al., 2020), HR 90-95% (Santos et al., 2022), HR >95% (Santos et al., 2022) |
| HRmax >85% TM-change |  |
| HRmax 75-85% %change | Percent playing time 75-84% HRMax (Castellano et al., 2015), Heart rate 65-85% Max (Hulka et al., 2016), Time spend 70-85% (%HRMax) (Aslan, 2013) |
| HRmax75-85% TM-change |  |
| HRmax <75% %change | Percent playing time <75% HRMax (Castellano et al., 2015), HR <75% (Castillo-Rodríguez et al., 2023), Heart rate <65% Max (Hulka et al., 2016), Time spend <70% (%HRMax) (Aslan, 2013), 75-84% HRMax (Casamichana & Castellano, 2010; Castillo-Rodríguez et al., 2023), 70-85% HRMax (Campos Vázquez et al., 2017), HR 60-70% (Santos et al., 2022), HR 70-80% (Santos et al., 2022) |
| HRmax <75% TM-change |  |
| Lactate %change* | Lactate (mmol/L) (Castagna et al., 2019), Blood lactate (mmol/l) (Cherni et al., 2025; Halouani et al., 2017a, 2017b; Rampinini et al., 2007), |
| Lactate TM-change* |  |
| Edwards’ TRIMP %change | Edwards' TRIMP (Espada et al., 2023; Fernando J. Santos et al., 2021), Trimp (Guard et al., 2022) |
| Edwards’ TRIMP TM-change |  |
| Player Load %change | Player Load (AU) (Casamichana et al., 2018; Castellano et al., 2015; Castellano et al., 2016; Gantois et al., 2023; Guard et al., 2022; Pantelić et al., 2019), Player Load Volume (AU) (Santos et al., 2024), Player Load (Intensity) (Santos et al., 2022; F. J. Santos et al., 2021), Players Load (Volume) (Santos et al., 2022; F. J. Santos et al., 2021) |
| Player Load TM-change |  |
| RPE %change* | RPE Global (Asian-Clemente et al., 2023; Aslan, 2013; Casamichana et al., 2018; Casamichana & Castellano, 2010; Castagna et al., 2019; Dalby, 2013; Dimitriadis et al., 2022; Guard et al., 2022; Halouani et al., 2017a, 2017b; Hulka et al., 2016; Köklü et al., 2013; Nunes et al., 2021; Nunes et al., 2020; Rampinini et al., 2007; Sampaio et al., 2007), RPE cardiorespiratory (Castagna et al., 2019), RPE muscle (Castagna et al., 2019) |
| RPE TM-change* |  |

Physical variables

| Variables in review | Variables in included studies |
| --- | --- |
| Total distance %change* | Total Distance (m) (Asian-Clemente et al., 2025; Asian-Clemente et al., 2023; Calderón Pellegrino et al., 2020; Campos Vázquez et al., 2017; Casamichana & Castellano, 2010; Castagna et al., 2019; Julen Castellano et al., 2017; Castellano et al., 2015; Castellano et al., 2016; Castillo-Rodríguez et al., 2023; Castillo et al., 2020a, 2020b; Cherni et al., 2025; Clemente et al., 2018; Dalby, 2013; Dimitriadis et al., 2022; Espada et al., 2023; Gantois et al., 2023; Goto & King, 2019; Guard et al., 2022; Hidalgo De Mora et al., 2024; Hodgson et al., 2014; Hulka et al., 2016; Joo et al., 2016; Lemes et al., 2020; Olthof et al., 2018; Pantelić et al., 2019; Sannicandro et al., 2020; Santos et al., 2024; Santos et al., 2022; Fernando Jorge Santos et al., 2021; Fernando J. Santos et al., 2021; F. J. Santos et al., 2021) |
| Total distance TM-change* |  |
| Relative total distance %change* | Total Distance (m/min) (Casamichana et al., 2018; Casamichana & Castellano, 2010; Castillo-Rodríguez et al., 2023; Dalby, 2013; Dimitriadis et al., 2022; Gantois et al., 2023; Guard et al., 2022), Relative Total Distance (m/min) (Campos Vázquez et al., 2017) |
| Relative total distance TM-change* |  |
| Max. speed %change* | Vpeak (km/h) (Cherni et al., 2025), Peak speed (km/h) (Asian-Clemente et al., 2025; Casamichana et al., 2018; Gantois et al., 2023; Pantelić et al., 2019), Vmax (km/h) (Campos Vázquez et al., 2017; Julen Castellano et al., 2017; Castellano et al., 2016; Hidalgo De Mora et al., 2024), VelocityMax (km/h) (Calderón Pellegrino et al., 2020; Castillo et al., 2020b), Max speed (km/h) (Casamichana & Castellano, 2010; Castillo-Rodríguez et al., 2023; Dalby, 2013; Guard et al., 2022; Nunes et al., 2021; Nunes et al., 2020; Sannicandro et al., 2020), Max. sprint (km/h) (Santos et al., 2022; Fernando Jorge Santos et al., 2021) |
| Max. speed TM-change* |  |
| Number of sprints %change* | Sprints (N) (Castillo et al., 2020b; Clemente et al., 2018; Nunes et al., 2021), Sprinting>16 km/h (N) (Castillo et al., 2020a), Sprint runs >20 km/h (N) (Pantelić et al., 2019) |
| Number of sprints TM-change |  |
| Sprinting distance %change | Sprinting >21 km/h (m) (Asian-Clemente et al., 2025; Calderón Pellegrino et al., 2020; Castillo-Rodríguez et al., 2023; Castillo et al., 2020b; Santos et al., 2024), Distance >21 km/h (m) (Julen Castellano et al., 2017; Guard et al., 2022), Sprinting distance >19,9 km/h (m) (Clemente et al., 2018), Distance covered >24 km/h (m) (Espada et al., 2023; Fernando J. Santos et al., 2021), Sprint running distance >25.2 km/h (m) (Olthof et al., 2018), Sprinting >20 km/h (m) (Pantelić et al., 2019) |
| Sprinting distance TM-change* |  |
| High-speed distance %change | High-intensity running distance >18 km/h (m) (Calderón Pellegrino et al., 2020), Zone 4: ≥ 18.0 km/h (m) (Cherni et al., 2025), running speed > 19.1 km/h (m)(Hidalgo De Mora et al., 2024), High speed running 18-21 km/h (m) (Calderón Pellegrino et al., 2020; Castillo-Rodríguez et al., 2023), Distance 17-21 km/h (m) (Julen Castellano et al., 2017), Distance covered 18-21 km/h (m) (Espada et al., 2023; Fernando J. Santos et al., 2021), High-intensity running distance >19.8 km/h (m) (Olthof et al., 2018), Running 19.9-25.2 km/h (m) (Sannicandro et al., 2020) |
| High-speed distance TM-change* |  |
| Running distance %change | Fast running 14-18 km/h (m) (Calderón Pellegrino et al., 2020; Cherni et al., 2025), Distance (m) medium-intensity running (13-18 km/h) (Casamichana & Castellano, 2010; Castillo-Rodríguez et al., 2023; Dalby, 2013), High-intensity running distance (m) >16 km/h (Castagna et al., 2019), Distance covered 13-16 km/h (Castellano et al., 2015), Distance 14-17 km/h (Julen Castellano et al., 2017), Cruising 14-21 km/h (m) (Castillo et al., 2020b), Cruising 13-16 km/h (m) (Castillo et al., 2020a),  Running distance 13,9-19,9 km/h (m)(Clemente et al., 2018), Distance 15-19 km/h (m) (Dimitriadis et al., 2022), Distance covered 12-18 km/h (m) (Espada et al., 2023; Gantois et al., 2023; Fernando J. Santos et al., 2021), Distance covered at speed >4.3 m/s (Goto & King, 2019), Distance covered 13-15.9 km/h (Joo et al., 2016), Distance at 14.3-21.4 km/h (Lemes et al., 2020), Distance covered 9-18 km/h (m) Running (Nunes et al., 2021), Distance covered >12 km/h (m) (Santos et al., 2022; Fernando Jorge Santos et al., 2021), Distance >13 km/h (Pantelić et al., 2019), Distance >16 km/h (Pantelić et al., 2019), Running distance 14.5-19.9 km/h (m) (Sannicandro et al., 2020) |
| Running distance TM-change |  |
| Jogging distance %change* | Running 7-14 km/h (m) (Calderón Pellegrino et al., 2020; Julen Castellano et al., 2017; Castillo et al., 2020b; Cherni et al., 2025), Distance (m) low-intensity running (7-13 km/h) (Casamichana & Castellano, 2010), Distance covered 8-13 km/h (Castellano et al., 2015; Castellano et al., 2016), Jogging 8-12.9 km/h (m) (Castillo et al., 2020a), Distance at 7-13 km/h (m) (Castillo-Rodríguez et al., 2023; Dalby, 2013), Distance 7-11 km/h (m) (Dimitriadis et al., 2022), Distance 11-15 km/h (m) (Dimitriadis et al., 2022), Distance covered 6-12 km/h (m) (Espada et al., 2023; Fernando J. Santos et al., 2021), Distance <11 km/h (m) (Guard et al., 2022), Distance covered 10-12.9 km/h (Joo et al., 2016), Distance at 7-14.3 km/h (Lemes et al., 2020), Running 9-18 km/h (Nunes et al., 2020), Running distance 7.3-14.5 km/h (m) (Sannicandro et al., 2020), Low-intensity running distance (<13 km/h) (Hidalgo De Mora et al., 2024) |
| Jogging distance TM-change |  |
| Walking distance %change* | Walking 0,5-7 km/h (m) (Calderón Pellegrino et al., 2020), Standing 0-0.5 km/h (m) (Calderón Pellegrino et al., 2020), Distance (m) Walking (0-7 km/h) (Casamichana & Castellano, 2010; Cherni et al., 2025), Distance covered <3 km/h (Castellano et al., 2015; Castillo et al., 2020a), Distance <8 km/h (m) (Castellano et al., 2016), Distance <7 km/h (Julen Castellano et al., 2017; Castillo-Rodríguez et al., 2023; Castillo et al., 2020b; Dalby, 2013), Low Walking <3 km/h (m) (Castillo et al., 2020a), Walking 3-8 km/h (m) (Castillo et al., 2020a), Walking distance < 6,88 km/h (m) (Clemente et al., 2018), Distance at 0-6.9 km/h (m) (Dimitriadis et al., 2022; Joo et al., 2016; Lemes et al., 2020), Distance covered 0-6 km/h (m) (Espada et al., 2023), Distance covered <9 km/h (m) Walking (Nunes et al., 2021; Nunes et al., 2020), Distance covered <6 km/h (m) (Fernando J. Santos et al., 2021), Running distance <7.3 km/h (m) (Sannicandro et al., 2020) |
| Walking distance TM-change |  |
| Acceleration %change | Acceleration 1-2.5 m/s/s (n) (Asian-Clemente et al., 2023; Castillo et al., 2020a), Acceleration >2.5 m/s/s (n) (Asian-Clemente et al., 2023; Cherni et al., 2025; Sannicandro et al., 2020), Number of Acceleration 2.5-3.5 m/s/s (Calderón Pellegrino et al., 2020), Number of Acceleration >3.5 m/s/s (Calderón Pellegrino et al., 2020), Accelerations (n) (Castillo et al., 2020a; Hidalgo De Mora et al., 2024; Sannicandro et al., 2020; Santos et al., 2022), Number (n) Medium Acceleration distance 2.5-4 m/s/s (Castillo et al., 2020a), Number (n) High Acceleration distance >4 m/s/s (Castillo et al., 2020a), Acceleration 1-2 m/s/s (n) (Dimitriadis et al., 2022; Gantois et al., 2023), Acceleration 2-3 m/s/s (n) (Dimitriadis et al., 2022; Gantois et al., 2023), Acceleration >3 m/s/s (n) (Asian-Clemente et al., 2025; Dimitriadis et al., 2022; Gantois et al., 2023), Acceleration >2.78 (n) (Guard et al., 2022), Moderate Accelerations (n) (Casamichana et al., 2018), High Accelerations (n) (Casamichana et al., 2018) |
| Acceleration TM-change* |  |
| Deceleration %change | Deceleration -1 to -2,5 m/s/s (n) (Asian-Clemente et al., 2023), Deceleration < -2,5 m/s/s (n) (Asian-Clemente et al., 2023; Sannicandro et al., 2020), Decelerations (n) (Castillo et al., 2020a; Hidalgo De Mora et al., 2024; Sannicandro et al., 2020; Santos et al., 2022; Fernando Jorge Santos et al., 2021), Number (n) Low Deceleration -1 to -2 m/s/s (Castillo et al., 2020a; Dimitriadis et al., 2022; Gantois et al., 2023), Number (n) Medium deceleration -2 to -4 m/s/s (Castillo et al., 2020a), Number (n) High Deceleration distance <-4 m/s/s (Castillo et al., 2020a), Deceleration < -3.0 (m/s/s) (Dimitriadis et al., 2022; Gantois et al., 2023), Deceleration -2 to -3 m/s/s (n) (Dimitriadis et al., 2022), Deceleration >-2,78 (n) (Gantois et al., 2023), Moderate Decelerations (n) (Casamichana et al., 2018), High Decelerations (n) (Casamichana et al., 2018), Deceleration < -3 m/s/s (n) (Asian-Clemente et al., 2025), Deceleration > -2.5 m/s/s (Cherni et al., 2025) |
| Deceleration TM-change |  |
| Distance Individual Speed %change | Distance <40% individual max speed (Julen Castellano et al., 2017; Castellano et al., 2015; Castellano et al., 2016), Distance >60% individual max speed (Julen Castellano et al., 2017; Castellano et al., 2015; Castellano et al., 2016), Distance 40-60% individual max speed (Julen Castellano et al., 2017; Castellano et al., 2015; Castellano et al., 2016) |
| Distance Individual Speed TM-change |  |
| HMLD %change* | High Metablic Load Distance >25.5 W/kg (Espada et al., 2023; Gantois et al., 2023; Santos et al., 2024), Distance high-power (m) 20-55 W/kg (Sannicandro et al., 2020), Distance (m) maximum-power >55 W/kg (Sannicandro et al., 2020) |
| HMLD TM-change* |  |
| HMLT Time %change | Time high-power (s) 20-55 W/kg (Sannicandro et al., 2020), Time (s) maximum power >55 W/kg (Sannicandro et al., 2020) |
| HMLT TM-change |  |
| COD %change | Moderate-intensity COD (n) (Casamichana et al., 2018), High-intensity COD (n) (Casamichana et al., 2018), High-intensity COD (n) (Casamichana et al., 2018) |
| COD TM-change |  |
| Work-to-Rest ratio %change* | Work-to-Rest Ratio (Campos Vázquez et al., 2017; Casamichana & Castellano, 2010; Julen Castellano et al., 2017; Castellano et al., 2015; Castellano et al., 2016), Work-to-Rest Ratio <11 km/h (Guard et al., 2022) |
| Work-to-Rest TM-change* |  |

Technical variables

| Variables in review | Variables in included studies |
| --- | --- |
| Ball touches %change | %Ball touches (Joo et al., 2016), Ball touches per possession (Massamba et al., 2020), Dominant foot (counts) (Nunes et al., 2021; Nunes et al., 2020), Non-Dominant foot (counts) (Nunes et al., 2021; Nunes et al., 2020), Number of receives (Kelly & Drust, 2009) |
| Ball touches TM-change |  |
| Ball possession %change | Number of ball possessions (n) (Cherni et al., 2025; Massamba et al., 2020), Ball possessions (Aslan, 2013; Olthof et al., 2018) |
| Ball possession TM-change |  |
| Passes %change | Accurate pass (Guven et al., 2016), Backwards pass (Joo et al., 2016), Forward pass (Joo et al., 2016), Forward pass 1/3 area (Joo et al., 2016), Long-distance pass (Joo et al., 2016),  Mid-distance pass (Joo et al., 2016), Number of passes (Kelly & Drust, 2009; Massamba et al., 2020), Passing number (Guven et al., 2016; Joo et al., 2016; Nunes et al., 2021; Nunes et al., 2020),  Short-distance pass (Joo et al., 2016), Side pass (Joo et al., 2016), Successful pass (Aslan, 2013; Cherni et al., 2025), Target pass (Kelly & Drust, 2009) |
| Passes TM-change |  |
| Dribbles %change | Control & Dribble (Casamichana & Castellano, 2010), Dribbling (Aslan, 2013), Number of dribbles (Hodgson et al., 2014; Kelly & Drust, 2009; Massamba et al., 2020) |
| Dribbles TM-change |  |
| Shots %change | Control & Shoot (Casamichana & Castellano, 2010), Goals/shot (Olthof et al., 2018), Number of shots (Hodgson et al., 2014), Shot(s) (Aslan, 2013; Guven et al., 2016; Kelly & Drust, 2009) |
| Shots TM-change |  |
| Turnover %change | Number lost balls (n) (Cherni et al., 2025), Interception (Casamichana & Castellano, 2010; Guven et al., 2016),Number of interceptions (Hodgson et al., 2014; Kelly & Drust, 2009),Transitions (Olthof et al., 2018), Unsuccessful pass (Aslan, 2013), Inaccurate pass (Guven et al., 2016) |
| Turnover TM-change |  |

Tactical variables

| Variables in review | Variables in included studies |
| --- | --- |
| Team width %change | Team width (J. Castellano et al., 2017; Praça et al., 2021), Team width attack (m) (Chung et al., 2019) |
| Team width TM-change |  |
| Team length %change | Team length (J. Castellano et al., 2017; Chung et al., 2019; Praça et al., 2021) |
| Team length TM-change |  |
| Surface area %change | Surface area difference (Frencken et al., 2013), Surface area (Olthof et al., 2018), Effective playing space (Silva et al., 2014; Silva et al., 2015) |
| Surface area TM-change |  |
| Stretch index %change | Stretch index total (Clemente et al., 2018), Stretch index goal-to-goal (Clemente et al., 2018), Stretch index goal-to-goal (Praça et al., 2021) |
| Stretch index TM-change |  |
| Inter-team distance %change | Inter-team distance (J. Castellano et al., 2017; Clemente et al., 2018; Olthof et al., 2018), Inter-team distance longitudinal (Frencken et al., 2013), Team separateness (Silva et al., 2014) |
| Inter-team distance TM-change |  |
| Length per width ratio %change | Length per Width (Olthof et al., 2018; Praça et al., 2021), Playing length per width ratio (Silva et al., 2014) |
| Length per width ratio TM-change |  |
| Spatial exploration index %change | Spatial exploration index (Clemente et al., 2018; Praça et al., 2021; Ueda et al., 2025), Spatial distribution variability (Entropy) (Silva et al., 2015) |
| Spatial exploration index TM-change |  |
