# Supplementary material for: The effect of relative pitch size on physiological, physical, technical and tactical variables in small-sided games: a literature review and practical guide
Source: Front Sports Act Living. 2025 May 6;7:1592536. doi: 10.3389/fspor.2025.1592536 (PMC12089100; doi:10.3389/fspor.2025.1592536)
Supplement: Supplementary file 3 [file Table3.docx]

| Reference | Sample | SSGs format | Pitch dimensions (Length x Width) | Relative pitch size | Regimen | Outcome variables | | | | |
| --- | --- | --- | --- | --- | --- | --- | --- | --- | --- | --- |
|  |  |  |  |  |  | Physiological | | Physical | Technical | Tactical |
| Asian-Clemente et al. (13) | N = 18  Professional academy players  16.1 ± 0.3 years | 3 vs 2 counter-attack | Size 1: 40 x 30 m  Size 2: 40 x 50 m  Size 3:40 x 70 m | Size 1: 240 m^2^  Size 2: 400 m^2^  Size 3: 560 m^2^ | 3 x 4 min/2 min passive recovery | - HRmax > 85%  - HRmax  - RPE | | - Acc.  - Dec.  - TD |  |  |
| Asian-Clemente et al. (48) | N = 25  Professional soccer players  21.9 ± 1.9 years | 9 vs 9 + 2 floaters | Size 1: 33.8 x 31 m  Size 2: 42 x 38.7 m  Size 3:48.8 x 46.5 m | Size 1: 50.8 m^2^  Size 2: 80.5 m^2^  Size 3: 115.9 m^2^ | 1 x 8 min |  | | - TD  - SD  - Max. Speed  - Acc.  - Dec. |  |  |
| Aslan (14) | N = 10  Recreational university players  31.7 ± 7.6 years | 5 vs 5 | Size 1: 44 x 23 m  Size 2: 57 x 30 m | Size 1: 101.2 m^2^  Size 2: 171 m^2^ | 1 x 40 min | - HRmax < 75%  - HRmax > 85%  - HRmax 75-85%  - HRmax  - HRmean  - RPE | |  | - Ball possessions  - Dribbles  - Passes  - Turnovers  - Shots |  |
|  |  | 7 vs 7 | Size 1: 44 x 23 m  Size 2: 57 x 30 m | Size 2: 72.3 m^2^  Size 2: 122.1 m^2^ | 1 x 40 min |  |  |  |  |  |
| Calderón Pellegrino et al. (97) | N = 16  Professional academy players  16.9 ± 0.3 years | 4 vs 4 | Size 1: n.a.  Size 2: n.a.  Size 3: n.a. | Size 1: 125 m^2^  Size 2: 150 m^2^  Size 3: 250 m^2^ | 1 x 8 min  (Fatigue inducing between sets) |  | | - JD  - HSRD  - RD  - SD  - WD  - Max. Speed  - Acc.  - Dec.  - TD |  |  |
| Campos Vázquez et al. (15) | N = 9  Professional soccer players  26.9 ± 3.7 years | 6 vs 6 + 1 floater | Size 1: 20 x 30 m  Size 2: 25 x 40 m  Size 3: 50 x 40 m | Size 1: 46.2 m^2^  Size 2: 76.9 m^2^  Size 3: 153.9 m^2^ | 4 x 5 min /2 min passive recovery | - HRmax > 85%  - HRmax < 75% | | - Max. Speed  - TD  - Rel. TD  - Work-to-Rest Ratio | - Turnovers  - Dribbles  - Shots |  |
| Casamichana & Castellano (16) | N = 10  Youth soccer players  15.5 ± 0.5 years | 5 vs 5 + GK | Size 1: 32 x 23 m  Size 2: 50 x 35 m  Size 3: 62 x 44 m | Size 1: 73.6 m^2^  Size 2: 175 m^2^  Size 3: 272.8 m^2^ | 3 x 8 min/5 min passive recovery | - HRmax > 85%  - HRmax < 75%  - HRmax  - HRmean  - RPE | | - JD  - RD  - WD |  |  |
| Casamichana et al. (31) | N = 20  Amateur soccer players  21 ± 5 years | 5 vs 5 + GK | Size 1: 40 x 25 m  Size 2: 66 x 25 m  Size 3: 50 x 40 m  Size 4: 66 x 50 m | Size 1: 100 m^2^  Size 2: 165 m^2^  Size 3: 200 m^2^  Size 4: 330 m^2^ | 4 x 6 min/8 min passive recovery | - HRmean  - PL  - RPE | | - COD  - Max. Speed  - Acc.  - Dec.  - Rel. TD |  |  |
| Castagna et al. (25) | N = 19  Professional academy-level soccer players  17.1 ± 0.3 years | 1 vs 1 | Size 1: 20 x 10 m  Size 2: 20 x 20 m  Size 3: 30 x 20 m | Size 1: 100 m^2^  Size 2: 200 m^2^  Size 3: 300 m^2^ | 4 x 2 min/3 min active recovery | - Lactate  - HRmax  - HRmean  - RPE | | - Acc.  - Dec.  - TD  - HMLD |  |  |
| Castellano et al. (17) | N = 24  Elite academy players  13.3 ± 0.5 years | 7 vs 7 | Size 1: 45 x 27 m  Size 2: 63 x38 m  Size 3: 78 x 46 m | Size 1: 100 m^2^  Size 2: 200 m^2^  Size 3: 300 m^2^ | 2 x 12 min/5 min passive recovery | - HRmax < 75 %  - HRmax > 85%  - HRmax 75-85%  - HRmax  - HRmean  - PL | | - JD  - RD  - SD  - WD  - TD  - Work-to-Rest Ratio |  |  |
|  |  | 9 vs 9 | Size 1: 52 x 31 m  Size 2: 73 x 44 m  Size 3: 90 x 45 m | Size 1: 100 m^2^  Size 2: 200 m^2^  Size 3: 300 m^2^ | 2 x 12 min/5 min passive recovery |  |  |  |  |  |
| Castellano et al. (24) | N = 44  Professional academy players  U12: 12.1 ± 0.4 years  U13: 13.3 ± 0.5 years | 7 vs 7 | Size 1: n.a.  Size 2: n.a.  Size 3: n.a. | Size 1: 100 m^2^  Size 2: 200 m^2^  Size 3: 300 m^2^ | 2 x 12 min/5 min passive recovery | - PL | | - JD  - RD  - SD  - WD  - Max. Speed  - TD  - Work-to-Rest Ratio |  |  |
|  |  | 9 vs 9 | Size 1: n.a.  Size 2: n.a.  Size 3: n.a. | Size 1: 100 m^2^  Size 2: 200 m^2^  Size 3: 300 m^2^ | 2 x 12 min/5 min passive recovery |  |  |  |  |  |
| Castellano et al. (61) | N = 13  Grasroots soccer academy  U13: 13.5 ± 0.3 years  U14: 14.3 ± 0.3 years | 6 vs 6 + GK | Size 1: 30 x 40 m  Size 2: 40 x 40 m  Size 3: 50 x 40 m  Size 4: 60 x 40 m | Size 1: 100 m^2^  Size 2: 133.3 m^2^  Size 3: 166.6 m^2^  Size 4: 200 m^2^ | 1 x 7 min/4 min passive recovery |  | |  |  | - Stretch index  - Team width  - Team length  - Inter-team distance |
| Castellano et al. (43) | N = 13  Grasroots soccer academy  U13: 13.5 ± 0.3 years  U14: 14.3 ± 0.3 years | 6 vs 6 + GK | Size 1: 30 x 40 m  Size 2: 40 x 40 m  Size 3: 50 x 40 m  Size 4: 60 x 40 m | Size 1: 100 m^2^  Size 2: 133.3 m^2^  Size 3: 166.6 m^2^  Size 4: 200 m^2^ | 1 x 7 min/4 min passive recovery | - PL | | - HSRD  - JD  - WD  - RD  - TD  - Max. Speed  - Work-to-Rest Ratio |  |  |
| Castillo et al. (54) | N = 20  National division players  14.9 ± 0.6 years | 5 vs 5 + GK | Size 1: 38 x 26 m  Size 2: 53 x 37 m | Size 1: 98.8 m^2^  Size 2: 196.1 m^2^ | 4 x 6 min/2 min recovery |  | | - SD  - RD  - TD  - Max. Speed |  |  |
| Castillo et al. (50) | N = 24  Performance academy players  11.8 ± 0.3 years | 6 vs 6 | Size 1: 22 x 13 m  Size 2: 32 x 19 m  Size 3: 39 x 23 m | Size 1: 25 m^2^  Size 2: 50 m^2^  Size 3: 75 m^2^ | 1 x 6 min |  | | - WD  - Acc.  - JD  - RD  - Dec.  - Number of sprints  - TD |  |  |
| Castillo et al. (51) | N = 20  Provincial division players  14.8 ± 0.6 years | 5 vs 5 + GK | Size 1: 38 x 26 m  Size 2: 53 x 37 m | Size 1: 100 m^2^  Size 2: 200 m^2^ | 4 x 6 min/2 min recovery | . | | - Dec.  - Max. Speed  - Acc.  - JD  - Number of sprints |  |  |
| Castillo-Rodríguez et al. (98) | N = 15  Regional level players  17.3 ± 0.5 years | 5 vs 5 + GK | Size 1: 33.8 x17.9 m  Size 2: 58.4 x 30.9 m  Size 3: 75.3 x 39.9 m | Size 1: 60.5 m^2^  Size 2: 180.46 m^2^  Size 3: 300.45 m^2^ | 3 x 8 min/5 min passive recovery | - HRmax < 75 %  - HRmax 75-85%  - HRmax > 85%  - HRmax  - HRmean | | - HSRD  - JD  - RD  - SD  - WD  - Max. Speed  - Rel. TD  - TD |  |  |
| Cherni et al. (26) | N = 36  Pre-PHV (14.7 ± 0.4 years)  Circa-PHV (14.4 ± 0.9 years)  Post-PHV (14.2 ± 0.7 years) | 3 vs 3 + GK  Size 1: 36 x 27 m | Size 2: 40 x 29 m  Size 1: 162 m^2^ | Size 2: 193.33 m^2^ | 2 x 4 min/1 min passive recovery | - HRmax  - HRmean  - Lactate  - RPE | | - TD  - Max. speed  - Acc.  - Dec.  - JD  - WD  - RD  - HSRD | - Passes  - Turnovers  - Ball possession |  |
|  |  | 4 vs 4 + GK | Size 1: 40 x 30 m  Size 2: 44 x 33 m | Size 1: 150 m^2^  Size 2: 181.5 m^2^ | 2 x 4 min/1 min passive recovery |  |  |  |  |  |
| Chung et al. (68) | N = 10  Regional level players  13.6 ± 0.5 years | 4 vs 4 + GK | Size 1: 40 x 30 m  Size 2: 44 x 33 m | Size 1: 150 m^2^  Size 2: 181.5 m^2^ | 2 x 4 min/1 min passive recovery |  | |  |  | - Team width  - Team length |
|  |  | 4 vs 4 |  | Size 2: 126 m^2^ |  |  |  |  |  |  |
|  |  | 5 vs 5 |  | Size 3: 100.8 m^2^ |  |  |  |  |  |  |
| Clemente et al. (55) | N = 10  Amateur soccer players  23.4 ± 3.9 years | 11 vs 11 | Size 1: 54 x 68 m  Size 2: 108 x 68 m | Size 1: 166.91 m^2^  Size 2: 333.82 m^2^ | 1 x 30 min |  | | - SD  - WD  - JD  - RD  - Number of sprints  - TD |  | - SEI |
| Clemente et al. (62) | N = 10  Amateur soccer players  23.4 ± 3.9 years | 11 vs 11 | Size 1: 54 x 68 m  Size 2: 108 x 68 m | Size 1: 166.91 m^2^  Size 2: 333.82 m^2^ | 1 x 30 min |  | |  |  | - Stretch index |
| Dalby (39) | N = 10  Collegiate soccer players  17 ± 1 years | 5 vs 5 + GK | Size 1: 48.5 x 32.4 m  Size 2: 62.1 x 41.4 m  Size 3: 73.2 x 48.8 m | Size 1: 157.14 m^2^  Size 2: 257.1 m^2^  Size 3: 357.22 m^2^ | 4 x 4 min/3 min recovery | - RPE | | - RD  - SD  - JD  - WD  - Max. Speed  - Rel. TD  - TD |  |  |
| Dimitriadis et al. (32) | N = 16  Active students  14.8 ± 0.5 years | 1 vs 1 + GK | Size 1: 10 x 15 m  Size 2: 20 x 10 m  Size 3: 20 x 15 m | Size 1: 75 m^2^  Size 2: 100 m^2^  Size 3: 150 m^2^ | 4 x 1 min/2 min recovery | - HRmean  - RPE | | - RD  - SD  - WD  - JD  - Acc.  - Dec.  - Rel. TD  - TD |  |  |
|  |  | 2 vs 2 + GK | Size 1: 20 x 15 m  Size 2: 27 x 15 m  Size 3: 30 x 20 m |  | 4 x 2 min/4 min recovery |  |  |  |  |  |
|  |  | 3 vs 3 + GK | Size 1: 25 x 18 m; Size 2: 30 x 20 m  Size 3: 36 x 15 m |  | 4 x 3 min/3 min recovery |  |  |  |  |  |
|  |  | 4 vs 4 + GK | Size 1: n. a.  Size 2: n. a.  Size 3: n. a. |  | n. a. |  |  |  |  |  |
| Espada et al. (46) | N = 24  Elite academy players (U12, U15)  Professional football players (U23)  U12: 11.7 ± 0.5 years  U15: 14.7 ± 0.8 years  U23: 20.1 ± 1.5 years | 4 vs 4 + GK | Size 1: 30 x 20 m  Size 2: 36 x 24 m | Size 1: 75 m^2^  Size 2: 108 m^2^ | 2 x 3 min/3 min recovery | - Edward’s TRIMP | - Dec.  - HSRD  - JD  - HMLD  - Acc.  - RD  - SD  - WD  - TD | |  |  |
| Frencken et al. (64) | N = 10  Amateur soccer players  22.3 ± 3 years | 4 vs 4 + GK | Size 1: 24 x 16 m  Size 2: 24 x 20 m | Size 1: 150 m^2^  Size 2: 300 m^2^ | 2 x 8 min/8 min recovery |  |  | |  | - Inter-team distance  - Surface area |
| Gantois et al. (45) | N = 12  First division academy players  17 ± 0.7 years | 3 vs 3 | Size 1: 20 x 15 m  Size 2: 30 x 20 m | Size 1: 50 m^2^  Size 2: 100 m^2^ | 4 x 4 min/ 2 min recovery |  | - JD  - RD  - Max. Speed  - HMLD  - PL  - HSRD  - Acc.  - Dec.  - Rel. TD  - TD | |  |  |
| Goto & King (56) | N = 11  Regional level players  16.3 ± 0.6 years | 6 vs 6 + GK | Size 1: 39 x 25 m  Size 2: 55 x 36 m  Size 3: 78 x 50 m | Size 1: 97.5 m^2^  Size 2: 198 m^2^  Size 3: 390 m^2^ | 1 x 35 min |  | - RD  - HMLD  - TD | |  |  |
| Guard et al. (19) | N = 12  Elite soccer players  18 ± 1.2 years | 6 vs 6 + GK | Size 1: 40 x 30 m  Size 2: 45 x 34 m  Size 3: 49 x 37 m | Size 1: 120 m^2^  Size 2: 153 m^2^  Size 3: 181.3 m^2^ | 4 x 4 min/2 min recovery | - Edward’s TRIMP  - HRmax > 85%  - HRmean  - PL  - RPE | - Acc.  - Dec.  - JD  - SD  - Max. Speed  - Rel. TD  - TD  - Work-to-Rest Ratio | |  |  |
| Guven et al. (60) | N = 8  Amateur players  27.2 ± 3.1 years | 4 vs 4 | Size 1: 26 x 24 m  Size 2: 40 x 30 m | Size 1: 78 m^2^  Size 2: 150 m^2^ | 3 x 6 min/5 min passive recovery |  |  | | - Dribbles  - Shots  - Passes  - Turnover  - Ball possession |  |
| Halouani et al. (33) | N = 16  Amateur players  13.2 ± 0.6 years | 4 vs 4 | Size 1: 15 x 10 m  Size 2: 20 x 15 m  Size 3: 25 x 20 m | Size 1: 18.75 m^2^  Size 2: 37.5 m^2^  Size 3: 62.5 m^2^ | 4 x 4 min/2 min passive recovery | - Lactate  - HRmean  - RPE |  | |  |  |
| Halouani et al. (34) | N = 18  Amateur players  13.5 ± 0.7 years | 2 vs 2 | 25 x 20 m | Size 1: 125 m^2^ | 4 x 4 min/2 min passive recovery | - Lactate  - HRmean  - RPE |  | |  |  |
|  |  | 3 vs 3 |  | Size 2: 83.33 m^2^ |  |  |  |  |  |  |
|  |  | 4 vs 4 |  | Size 3: 62.5 m^2^ |  |  |  |  |  |  |
| Hidalgo De Mora et al. (52) | N = 14  U18 players  17.1 ± 0.6 years | 7 v 7 + GK | Size 1: 40 x 34 m  Size 2: 68 x 40 m | Size 1: 97.14 m^2^  Size: 194.26 m^2^ | 6 x 4 min/2 min recovery |  | - TD  - WD  - RD  - SD  - Max. Speed  - Acc.  - Dec. | |  |  |
| Hodgson et al. (35) | N = 8  University-level players  20 ± 1 years | 5 vs 5 + GK | Size 1: 30 x 20 m  Size 2: 40 x 30 m  Size 3: 50 x 40 m | Size 1: 60 m^2^  Size 2: 120 m^2^  Size 3: 200 m^2^ | 4 x 4 min/3 min recovery | - HRmean | - HSRD  - SD  - TD  - Acc.  - Dec. | | - Shots  - Turnovers  - Passes  - Dribbles |  |
| Hulka et al. (20) | N = 29  Junior soccer players  18.11 ± 1.31 years | 5 vs 5 + GK | Size 1: 28 x 20 m  Size 2: 35 x 25 m  Size 3: 42 x 30 m | Size 1: 56 m^2^  Size 2: 87.5 m^2^  Size 3: 126 m^2^ | 3 x 4 min/3 min recovery | - RPE  - HRmax < 75%  - HRmax 75 – 85%  - HRmax > 85%  - HRmean | - TD | |  |  |
| Joo et al. (99) | N = 149  Young players  12 ± 0.4 years | 7 vs 7 + GK | Size 1: 68 x 47 m  Size 2: 75 x 47 m | Size 1: 228.29 m^2^  Size 2: 251. 79 m^2^ | 1 x 30 min |  | - SD  - WD  - HSRD  - JD  - RD  - TD | | - Passes  - Shots  - Ball touches |  |
| Kelly & Drust (100) | N = 8  Professional players  18 ± 1 years | 4 vs 4 + GK | Size 1: 30 x 20 m  Size 2: 40 x 30 m  Size 3: 50 x 40 m | Size 1: 60 m^2^  Size 2: 120 m^2^  Size 3: 200 m^2^ | 4 x 4 min/ 2 min active recovery | - HRmean |  | | - Turnovers  - Passes  - Dribbles  - Shots |  |
| Köklü et al. (27) | N = 16  Elite academy players  14.2 ± 0.6 years | 3 vs 3 | Size 1: 20 x 15 m; Size 2: 25 x 18 m Size 3: 30 x 20 m | Size 1: 50 m^2^  Size 2: 75 m^2^  Size 3: 100 m^2^ | 4 x 4 min/ 2 min passive recovery | - HRmax  -Leme HRmean  - RPE |  | |  |  |
|  |  | 4 vs 4 | Size 1: 20 x 20 m; Size 2: 30 x 20 m Size 3: 32 x 25 m |  | 4 x 3 min/2 min passive recovery |  |  |  |  |  |
| Lemes et al. (58) | N = 48  National competitive players  U13 & U14 | 3 vs 3 + 1 + GK | Size 1: 36 x 27 m  Size 2: 40 x 29 m | Size 1: 138.86 m^2^  Size 2: 165.71 m^2^ | 4 x 4 min/4 min passive recovery |  | - RD  - JD  - WD  - TD | |  |  |
| Massamba et al. (21) | N = 10  Elite players  13 ± 0.3 years | 5 vs 5 | Size 1: 30 x 20 m  Size 2: 42 x 28 m  Size 3: 51 x 34 m | Size 1: 60 m^2^  Size 2: 117.6 m^2^  Size 3: 173.4 m^2^ | 4 x 4 min/1 min passive recovery | - HRmax > 85%  - HRmean |  | | - Ball touches  - Ball possessions  - Dribbles  - Passes |  |
| Nunes et al. (40) | N = 20  University-level players  22.3 ± 2 years | 4 vs 2 | 30 x 25 m | Size 1: 125 m^2^ | 4 x 4 min/4 min recovery | - RPE | - SD  - WD  - JD  - Max. Speed | | - Passes |  |
|  |  | 4 vs 3 |  | Size 2: 107.14 m^2^ |  |  |  |  |  |  |
|  |  | 4 vs 4 |  | Size 3: 93.76 m^2^ |  |  |  |  |  |  |
|  |  | 4 vs 5 |  | Size 4: 83.33 m^2^ |  |  |  |  |  |  |
|  |  | 4 vs 6 |  | Size 5: 75 m^2^ |  |  |  |  |  |  |
| Nunes et al. (41) | N = 52  Three football teams  U11: 10 ± 0.7 years  U15: 14 ± 1.3 years  U23: 21 ± 1.6 years | 4 vs 4 | Size 1: 20 x 15 m  Size 2: 25 x 20 m  Size 3: 30 x 25 m | Size 1: 37.5 m^2^  Size 2: 62.5 m^2^  Size 3: 93.8 m^2^ | 4 x 4 min/4 min active recovery | - RPE | - SD  - WD  - RD  - Max. Speed  - Number Sprints | | - Passes |  |
| Nunes et al. (101) | N = 20  University-level players  22.3 ± 2 years | 4 vs 3 | Size 1: 20 x 15 m  Size 2: 25 x 20 m  Size 3: 30 x 25 m | Size 1: 42.86 m^2^  Size 2: 71.43 m^2^  Size 3: 107.14 m^2^ | 4 x 4 min/4 min active recovery | - RPE | - SD  - WD  - JD  - Max. Speed | | - Passes |  |
|  |  | 4 vs 4 |  | Size 1: 37.5 m^2^  Size 2: 62.5 m^2^  Size 3: 93.8 m^2^ |  |  |  |  |  |  |
|  |  | 4 vs 5 |  | Size 1: 33.33 m^2^  Size 2: 55.55 m^2^  Size3: 83.33 m^2^ |  |  |  |  |  |  |
| Olthof et al. (59) | N = 125  Three professional academies  U13: 12.5 ± 0.5 years  U15: 14.4 ± 0.5 years  U17: 16.6 ± 3.2 years  U19: 17.9 ± 1 years | 4 vs 4 + GK | Size 1: 40 x 30 m  Size 2: 68 x 47 m | Size 1: 150 m^2^  Size 2: 399.5 m^2^ | 5 x 4 min/4 min recovery |  | - HSRD  - SD  - TD | | - Ball possessions  - Shots | - Inter-team distance  - Stretch index  - Surface area  - Length per width ratio |
| Owen et al. (28) | N = n .a.  English professional players  17.5 ± 1.1 years | 1 vs 1 | Size 1: 10 x 5 m  Size 2: 15 x 10 m  Size 3: 20 x 15 m | Size 1: 25 m^2^  Size 2: 75 m^2^  Size 3: 150 m^2^ | 3 x 3 min/12 min active recovery | - HRmax  - HRmean |  | |  |  |
|  |  | 2 vs 2 | Size 1: 15 x 10 m; Size 2: 20 x 15 m  Size 3: 25 x 20 m | Size 1: 37.5 m^2^  Size 2: 75 m^2^  Size 3: 125 m^2^ |  |  |  |  |  |  |
|  |  | 3 vs 3 | Size 1: 20 x 15 m  Size 2: 25 x 20 m  Size 3: 30 x 25 m | Size 1: 50 m^2^  Size 2: 83.3m^2^  Size 3: 125 m^2^ |  |  |  |  |  |  |
|  |  | 4 vs 4 | Size 1: 25 x 20 m  Size 2: 30 x 25 m  Size 3: 35 x 30 m | Size 2: 62.5 m^2^  Size 2: 93.8 m^2^  Size 3: 131.3 m^2^ |  |  |  |  |  |  |
|  |  | 5 vs 5 | Size 1: 30 x 25 m Size 2: 35 x 30 m  Size 3: 40 x 35 m | Size 1: 75 m^2^  Size 2: 105 m^2^  Size 3: 140 m^2^ |  |  |  |  |  |  |
| Pantelić et al. (22) | N = 10  Recreational players  20.1± 1.1 years | 4 vs 4 + GK | Size 1: 35 x 17 m  Size 2: 40 x 20 m | Size 1: 59.5 m^2^  Size 2: 80 m^2^ | 2 x 20 min/5 min passive recovery | - HRmax > 85%  - HRmax  - HRmean  - PL | - RD  - SD  - Max. Speed  - Number Sprints | |  |  |
| Praca et al. (63) | N = 48  National level youth academy  U13: 13.1 ± 0.6 years  U14: 14.3 ± 0.7 years | 3 vs 3 + 1 + GK | Size 1: 36 x 27 m  Size 2: 40 x 29 m | Size 1: 138.9 m^2^  Size 2: 165.7 m^2^ | 4 x 4 min/ 4 min passive recovery |  |  | |  | - Stretch index  - Length per width ratio  - SEI |
| Rampini et al. (37) | N = 20  Amateur players  24.5 ± 4.1 years | 3 vs 3 | Size 1: 20 x 12 m  Size 2: 25 x 15 m  Size 3: 30 x 18 m | Size 1: 40 m^2^  Size 2: 50 m^2^  Size 3: 90 m^2^ | 3 x 4 min/3 min active recovery | - Lactate  - HRmean  - RPE |  | |  |  |
|  |  | 4 vs 4 | Size 1: 24 x 16 m  Size 2: 30 x 20 m  Size 3: 36 x 24 m | Size 1: 48 m^2^  Size 2: 75 m^2^  Size 3: 108 m^2^ |  |  |  |  |  |  |
|  |  | 5 vs 5 | Size 1: 28 x 20 m  Size 2: 35 x 25 m  Size 3: 42 30 m | Size 1: 56 m^2^  Size 2: 87.5 m^2^  Size 3: 126 m^2^ |  |  |  |  |  |  |
|  |  | 6 vs 6 | Size 1: 32 x 24 m  Size 2: 40 x 30 m  Size 3: 48 x 36 m | Size 1: 64 m^2^  Size 2: 100 m^2^  Size 3: 144 m^2^ |  |  |  |  |  |  |
| Sampaio et al. (38) | N = 8  National standard players  15 ± 0 years | 2 vs 2 | 30 x 20 m | Size 1: 150 m^2^ | 2 x 3 min/90 sec recovery | - HRmean  - RPE |  | |  |  |
|  |  | 3 vs 3 |  | Size 2: 100 m^2^ |  |  |  |  |  |  |
| Sannicandro et al. (53) | N = 24  Professional soccer players  24.7 ± 3.9 years | 5 vs 5 + GK | 60 x 35 m | Size 1: 210 m^2^ | 4 x 3 min/1 min passive recovery |  | - WD  - HMLD  - HSRD  - JD  - RD  - Max. Speed  - Acc.  - Dec.  - TD | |  |  |
|  |  | 6 vs 6 + GK |  | Size 2: 175 m^2^ |  |  |  |  |  |  |
|  |  | 7 vs 7 + GK |  | Size 3: 150 m^2^ |  |  |  |  |  |  |
| Santos et al. (29) | N = 16  Regional and national players  U12 & U15 | 4 vs 4 | Size 1: 24 x 16 m | 48 m^2^ | 5 x 3 min/3 min recovery | - HRmax  - HRmean | - RD  - Max. Speed  - Acc.  - Dec.  - TD | |  |  |
|  |  | 4 vs 4 | Size 2: 30 x 20 m | 75 m^2^ |  |  |  |  |  |  |
|  |  | 4 vs 4 + GK | Size 3: 30 x 20 m | 75 m^2^ |  |  |  |  |  |  |
|  |  | 4 vs 4 | Size 4: 36 x 24 m | 108 m^2^ |  |  |  |  |  |  |
|  |  | 4 vs 4 + GK | Size 5: 36 x 24 m | 108 m^2^ |  |  |  |  |  |  |
| Santos et al. (47) | N = 24  Regional and national players  U12: 11.7 ± 0.5 years  U15: 14.7 ± 0.8 years  U23: 20.1 ± 1.5 years | 4 vs 4 | Size 1: 24 x 16 m  Size 2: 30 x 20 m  Size 3: 36 x 24 m | Size 1: 48 m^2^  Size 2: 75 m^2^  Size 3: 108 m^2^ | 3 x 3 min/3 min recovery | - Edward’s TRIMP | - JD  - RD  - HMLD  - HSRD  - SD  - WD  - TD | |  |  |
| Santos et al. (30) | N = 10  Professional sub-elite players  20.1 ± 1.5 years | 4 vs 4 | Size 1: 24 x 16 m | 48 m^2^ | 5 x 3 min/3 min recovery | - HRmax  - HRmean  - PL |  | |  |  |
|  |  | 4 vs 4 | Size 2: 30 x 20 m | 75 m^2^ |  |  |  |  |  |  |
|  |  | 4 vs 4 + GK | Size 3: 30 x 20 m | 75 m^2^ |  |  |  |  |  |  |
|  |  | 4 vs 4 | Size 4: 36 x 24 m | 108 m^2^ |  |  |  |  |  |  |
|  |  | 4 vs 4 + GK | Size 5: 36 x 24 m | 108 m^2^ |  |  |  |  |  |  |
| Santos et al. (23) | N = 10  Regional championship players  11.7 ± 0.5 years | 1 vs 1 | Size 1: 10 x 5 m  Size 2: 15 x 10 m  Size 3: 20 x 15 m | Size 1: 25 m^2^  Size 2: 75 m^2^  Size 3: 150 m^2^ | 3 x 2 min/3 min recovery | - HRmax < 75%  - HRmax > 85%  - HRmax  - HRmean  - PL | - RD  - Max. Speed  - Acc.  - Dec.  - TD | |  |  |
|  |  | 1 vs 1 + 1 | Size 1: 10 x 5 m  Size 2: 15 x 10 m  Size 3: 20 x 15 m | Size 1: 16.66 m^2^  Size 2: 50 m^2^  Size 3: 100 m^2^ |  |  |  |  |  |  |
| Santos et al. (44) | N = 24  Regional and national players  U12: 11.7 ± 0.5 years  U15: 14.7 ± 0.8 years  U23: 20.1 ± 1.5 years | 4 vs 4 | Size 1: 24 x 16 m | 48 m^2^ | 5 x 3 min/3 min recovery | - PL | - Acc.  - Dec.  - HMLD  - SD  - TD | |  |  |
|  |  | 4 vs 4 | Size 2: 30 x 20 m | 75 m^2^ |  |  |  |  |  |  |
|  |  | 4 vs 4 + GK | Size 3: 30 x 20 m | 75 m^2^ |  |  |  |  |  |  |
|  |  | 4 vs 4 | Size 4: 36 x 24 m | 108 m^2^ |  |  |  |  |  |  |
|  |  | 4 vs 4 + GK | Size 5: 36 x 24 m | 108 m^2^ |  |  |  |  |  |  |
| Silva et al. (65) | N = 20  Regional and national youth players from two teams  Team A: 16.2 ± 0.6 years  Team B: 15.6 ± 0.5 years | 4 vs 4 + GK | Size 1: 36.8 x 23.8 m  Size 2: 47.3 x 30.6 m  Size 3: 57.8 x 37.4 m | Size 1: 62.6 m^2^  Size 2: 103.4 m^2^  Size 3: 154.4 m^2^ | 3 x 7 min/7 min recovery |  |  | |  | - Length per width ratio  - Surface area  - Inter-team distance |
| Silva et al. (66) | N = 24  Regional level players  14.5 ± 0.5 years | 6 vs 6 | Size 1: 52.9 x 34.4 m  Size 2: 49.5 x 32.2 m  Size 3: 46.7 x 30.3 m | Size 1: 117.9 m^2^  Size 2: 132.8 m^2^  Size 3: 151.6 m^2^ | 3 x 6 min/4 min recovery |  |  | |  | - Surface area  - SEI |
| Ueda et al. (67) | N = 36  Professional youth soccer players  U10 (10.28 ± 0.58)  U11 (11.4 ± 0.27) | 4 vs 4 + GK | Size 1: 26.57 x 19.51 m  Size 2: 54.56 x 40 m | Size 1: 64.8 m^2^  Size 2: 272.8 m^2^ | n. a. |  |  | |  | - SEI |
|  |  | 5 vs 5 + GK | Size 1: 29.74 x 21.79 m  Size 2: 61 x 44.72 m | Size 1: 64.8 m^2^  Size 2: 272.8 m^2^ | n. a. |  |  |  |  |  |

Abbreviations: Acc. – Acceleration, Dec. – Deceleration, HMLD – High metabolic load distance, HSRD – High speed running distance, JD – Jogging distance, PL – Player Load, RD – Running distance, RPE – rating of perceived exertion, SD – Sprint distance, SEI – spatial exploration index, TD – Total distance, TRIMP – Training impulse, WD – Walking distance
